# A functional metagenomics study of soil carbon and nitrogen degradation networks and limiting factors on the Tibetan plateau

**DOI:** 10.3389/fmicb.2023.1170806

**Published:** 2023-05-05

**Authors:** Chong Yang, Hong Zhang, Xinquan Zhao, Pan Liu, Lushan Wang, Wenying Wang

**Affiliations:** ^1^School of Geographical Sciences, Qinghai Normal University, Xining, China; ^2^School of Life Sciences, Qinghai Normal University, Xining, China; ^3^State Key Laboratory of Microbial Technology, Shandong University, Qingdao, China; ^4^Northwest Plateau Institute of Biology, Chinese Academy of Sciences, Xining, China

**Keywords:** alpine grasslands, soil microorganisms, metagenomics, organic carbon degradation network, nitrogen cycle

## Abstract

**Introduction:**

The Three-River Source Nature Reserve is located in the core area of the Qinghai-Tibetan Plateau, with the alpine swamp, meadow and steppe as the main ecosystem types. However, the microbial communities in these alpine ecosystems, and their carbon and nitrogen degrading metabolic networks and limiting factors remain unclear.

**Methods:**

We sequenced the diversity of bacteria and fungi in alpine swamps, meadows, steppes, and their degraded and artificially restored ecosystems and analyzed soil environmental conditions.

**Results:**

The results indicated that moisture content had a greater influence on soil microbial community structure compared to degradation and restoration. Proteobacteria dominated in high moisture alpine swamps and alpine meadows, while Actinobacteria dominated in low moisture alpine steppes and artificial grasslands. A metabolic network analysis of carbon and nitrogen degradation and transformation using metagenomic sequencing revealed that plateau microorganisms lacked comprehensive and efficient enzyme systems to degrade organic carbon, nitrogen, and other biological macromolecules, so that the short-term degradation of alpine vegetation had no effect on the basic composition of soil microbial community. Correlation analysis found that nitrogen fixation was strong in meadows with high moisture content, and their key nitrogen-fixing enzymes were significantly related to *Sphingomonas*. Denitrification metabolism was enhanced in water-deficient habitats, and the key enzyme, nitrous oxide reductase, was significantly related to *Phycicoccus* and accelerated the loss of nitrogen. Furthermore, *Bacillus* contained a large number of amylases (GH13 and GH15) and proteases (S8, S11, S26, and M24) which may promote the efficient degradation of organic carbon and nitrogen in artificially restored grasslands.

**Discussion:**

This study illustrated the irrecoverability of meadow degradation and offered fundamental information for altering microbial communities to restore alpine ecosystems.

## 1. Introduction

Located in the hinterland of the Tibetan Plateau, the Three-river Source Region is the source of the Yangtze, Yellow, and Mekong Rivers, as well as a significant water conservation area in China and around the world (Liu et al., 2019). The region is rich in alpine grassland resources and play an important role in conserving water resources, regulating climate, protecting species diversity, maintaining ecological balance, and sustaining a developing animal husbandry economy (Qian et al., 2021). However, due to global climate change (i.e., accelerated freezing and thawing) and human activities (overgrazing) (Shang et al., 2008; Lin et al., 2015), acting in concert with the fragility and sensitivity of alpine grassland ecosystems, the region is suffering decreased grassland biodiversity, deterioration of plant productivity, destruction of soil physical structure, and continuous deterioration of the ecological environment (Zhou et al., 2019; Dong et al., 2021). There are about 0.45 × 10^8^ hm^2^ of degraded grassland on the Tibetan Plateau, accounting for about one-third of the total alpine grassland area (Wang Y. et al., 2021). Slightly and moderately degraded grasslands can be restored to their original state within 10–20 years by reducing grazing pressure and controlling rodent damage, but severely degraded alpine grasslands can only be restored using artificial vegetation restoration strategies (Feng et al., 2009). The establishment of artificial grassland can increase vegetation cover and improve soil quality.

Soil microorganisms are an important biological feature of soil quality and play a crucial role in grassland succession (Wang D. et al., 2022). Degradation of alpine grasslands decreases plant diversity and biomass both above and below ground, affects soil nutrient cycling, and causes soil microbial changes (Wu et al., 2014; Hirsch et al., 2016; Pan et al., 2018; Peng et al., 2020). Different microorganisms play specific roles in soil material circulation processes, such as decomposing litter and symbiotic relationships with plant roots (Matulich and Martiny, 2015; Kytöviita and Vestberg, 2020). As a result, the diversity of soil microbial community structures, as well as the function of their extracellular enzymes, is critical to the study of vegetation and soil ecology and ecological balance. Previous studies have found no significant differences in bacterial alpha-diversity among different degraded alpine grasslands on the Tibetan Plateau (Zhou et al., 2019), while alpine meadow degradation increases fungal diversity, suggesting that fungal communities are more sensitive to grassland degradation than are bacterial communities (Che et al., 2019; Dong et al., 2021). While previous studies (Cao et al., 2022; Wang X. et al., 2022) have focused in taxonomic diversity and community composition, functional studies dealing with carbon and nitrogen degrading systems are largely unknown in this area of the planet.

Alpine swamp, meadow and steppe are the main ecosystem types in the Three-river Source Region, and are sensitive to global climate change (Li Y. M. et al., 2019; Peng et al., 2020). The permafrost regions of the Tibetan Plateau store a large amount of organic carbon and nitrogen, but climate warming and grassland degradation will cause the loss of these carbon and nitrogen reservoirs (Baumann et al., 2009; Li L. H. et al., 2019). Climate warming causes the permafrost melting and stimulates the decomposition of more recalcitrant and inert carbon by microorganisms (Liu et al., 2020; Chen et al., 2021). The soils of degraded grasslands on the Tibetan Plateau release CO_2_ and N_2_O as greenhouse gases, thereby decreasing soil carbon and nitrogen pools (Wen et al., 2012). Microorganisms in the plateau environment also have low nitrogen mineralization rates and available phosphorus release, which limits the growth of plants and microorganisms according to the available nitrogen and phosphorus (Mao et al., 2020; Yang et al., 2021). Soil microorganisms are the main regulators of the earth’s carbon, nitrogen, and phosphorus cycles. At present, it remains unclear what effect degraded and artificially restored grasslands will have on changes in microbial communities and functional diversity. It is also unknown whether there is a relationship between the functional diversity of microorganisms, soil nutrients and plant communities. The use of metagenomics approach to locate relevant key microorganisms and key enzymes, map microbial networks and their metabolic pathways, and reveal the functional diversity of microbial metabolic networks would help find the limiting factors of carbon and nitrogen degradation in alpine grasslands, thus better understanding the degradation mechanisms in low-temperature habitats and providing new perspectives for ecological conservation and restoration.

Using second generation high-throughput sequencing technology, this study investigated plant communities, soil nutrients, and the structure and function of soil microbial communities in the three types of native alpine grasslands with different soil water contents (i.e., alpine swamps wetland, alpine meadow, and dry alpine steppe) on the Tibetan Plateau. The effects of vegetation degradation and artificial restoration measures in alpine meadows and steppes on soil nutrients and the structure and function of soil microbial communities were evaluated to reveal the characteristics and metabolic types of different alpine grassland ecosystems. By analyzing the responses of soil physicochemical properties, soil microbial communities, and the abundance of genes related to carbon and nitrogen cycles to degradation and restoration, we revealed how degradation, and artificial restoration in grasslands affected soil nutrition, microbial community dynamics and functional changes through changes in nutrient utilization. Our results provided an important theoretical basis for understanding the degradation and restoration mechanisms of alpine grasslands on the Tibetan Plateau.

## 2. Materials and methods

### 2.1. Overview of the study area and field sampling

The study is carried out in Maduo County and Maqin County in the Guoluo Tibetan Autonomous Prefecture of Qinghai Province, China (Supplementary Figure S1). The geographical coordinates of Maqin study sites are between 100°13′ E–100°30′ E and 34°21’ N–34°28’ N, with an average altitude of 3,700–3,960 m. The cold season lasts for 7–8 months in this region, with an annual average temperature of 0.7°C and an annual average precipitation of 524 mm from 2000 to 2018 year. The native vegetation types in this study site are mainly alpine meadow (dominated by *Kobresia humilis* and *Kobresia capillifolia*) and alpine swamp (dominated by *Kobresia tibetica*). The geographical coordinates of Maduo study sites is between 98°14′ E–98°26′ E and 34°50’ N–34°52’ N, with an altitude of 4,200 m. The cold season lasts for 8–9 months in this region, with an annual average temperature of −2.6°C and an annual average precipitation of 352 mm from 2000–2018 year. The vegetation types in this study site are mainly alpine steppe and dominated by *Stipa purpurea*. Samples were collected during the growing season in mid-August 2017.

The 10 sites in this study (Supplementary Table S1) are representative of the 3 main natural grasslands, such as alpine swamp G1, alpine meadow G2, and alpine steppe G8, 2 degraded alpine grasslands such as severely degraded alpine meadow G3 and degraded alpine steppe G9, no severely degraded alpine swamp at study site, and 5 artificial restored grasslands G4, G5, G6, G7, G10 (planted on heavily degraded grasslands with different restored year). Each sampling site includes the 3 repeated sample plots of 10 m^2^ that are as close to each other as possible, so that they have similar topography, vegetation, and soil types. The severely degraded grasslands were dominated by *Lancea tibetica, Artemisia hedinii, Potentilla anserina, Leontopodium nanum*, and total vegetation cover was <40%. The artificial restored grasslands were that severely degraded lands planted with well-adapted perennial grasses such as *Elymus sikiricus, Poa crymophila,* then managed artificially. All plots except G4 were grazing in winter with 50–70% forage utilization rate.

Three 10 m × 10 m plots were randomly deployed at each site. Three 1 m × 1 m samples in each 100 m^2^ plot were used for sample collection, and the above-ground parts of all plants were collected and dried to estimate the above-ground biomass (AB). In each sample, three random soil cores (0–10 cm) were collected using a 5-cm diameter soil auger into sterilized bags with irradiation treatments placed in a refrigerated foam box for immediate shipment back to the laboratory. A 2 mm soil sieve was used to separate the roots and soil. The separated roots were cleaned and dried to estimate underground biomass (UB). The sieved soil was divided into two parts: one was stored in a refrigerator at −20°C for microbial studies, and the other was stored at 4°C for the determination of soil physiochemical properties.

### 2.2. Determination of soil physiochemical properties

Soil moisture content (MC) was measured by the gravimetric method. Soil bulk density (BD) was measured by the cutting-ring method (Zhou et al., 2019). The pH value was measured using an acidity meter (FiveEasy Plus FE28, Mettler Toledo, Shanghai, China). Total carbon (TC), total nitrogen (TN), and total organic carbon (TOC) were measured using an Elementar Vario ELIII elemental analyzer (Germany). Soil available nitrogen (soluble organic nitrogen, SON; ammonium nitrogen, NH_4_^+^-N; nitrate nitrogen, NO_3_^−^-N), available phosphorus (AP), and total phosphorus (TP) were determined using a CleverChem Anna automatic discontinuous chemical analyzer (FUTURA, France). The soil SON content is soluble total nitrogen content subtracted by NH_4_^+^-N and NO_3_^−^-N content. Soil available potassium (AK) and total potassium (TK) were determined using an atomic absorption spectrometer (Shimadzu A6300, Japan).

### 2.3. DNA extraction and amplicon sequencing

A DNA extraction kit (Omega Bio-tek, Norcross, GA, United States) was used to extract total DNA from the 30 soil samples, according to the manufacturer’s instructions. The purity and quality of DNA were detected using a NanoDrop ND-1000 spectrophotometer (Thermo Fisher Scientific Inc., Waltham, MA, United States) and 1.2% agarose gel electrophoresis. DNA samples were stored at −20°C. The extracted DNA was subjected to high-throughput sequencing using an Illumina Miseq platform (Nanjing Shendu Life Technology Co., Ltd., Nanjing, China). Specifically, 338F (5’-ACTCCTACGGGAGGCAGCAG-3′) and 806R (5’-GGACTACHVGGGTWTCTAAT-3′) were used to perform amplicon sequencing of soil DNA against the V3 and V4 regions of 16S rRNA gene, using the primers ITS1 (5’-TCCGTAGGTGAACCTGCGG-3′) and ITS4 (5’-TCCTCCGCTTATTGATATGC-3′) to amplify fungal diversity against internal transcribed spacers of ITs rRNA gene. PCR amplification proceeded as follows: initial denaturation at 95°C (3 min), followed by 30 cycles of 95°C denaturation (30s), 55°C annealing (30s), 72°C extension (45 s), and finally 72°C extension (10 min) ending at 4°C. PCR reactions were performed in triplicate 20 μl mixture containing 4 μl of 5 × FastPfu Buffer, 2 μl of 2.5 mM dNTPs, 0.8 μl of each primer (5 μM), 0.4 μl of FastPfu Polymerase and 10 ng of template DNA. The PCR products were purified using an AxyPrep DNA Gel Extraction Kit (Axygen Biosciences, Union City, CA, United States). The purified amplicons were paired and sequenced using the Illumina MiSeq platform (Illumina, San Diego, CA, United States).

FLASH was used to filter and merge the rawfastq files of the 30 alpine grassland soil samples (Magoc and Salzberg, 2011). UPARSE version 7.1^1^ was used to cluster optimized sequences with 97% similarity into operational taxonomic units (OTUs). The UCHIME method was used to identify and remove chimeric OTUs (Edgar, 2013). The Ribosomal Database Project (RDP) Classifier version 2.2 (Wang et al., 2007) was used for 16S rRNA database^2^ and ITs rRNA database^3^ classification analysis, with a confidence threshold of 0.7.

### 2.4. Metagenomics

The three biomass replicates from each sampling site were mixed together to form 10 representative samples. The Illumina HiSeqXten platform was used for metagenomic sequencing (Shanghai Meiji Biomedical Technology Co., Ltd.) and the volume of sequencing data for each sample was 12 Gbase. Fastp was used to remove low-quality reads (length < 50 bp or with a quality value <20 or having N bases) (Chen et al., 2018). MEGAHIT was used to assemble contigs with length ≥ 300 bp (Li et al., 2015). Then the contigs were predicted with MetaGene (Noguchi et al., 2006) and clustered with parameters of 90% identity and 90% coverage to construct a non-redundant gene set using CD-HIT (Fu et al., 2012). Diamond was used to compare the amino acid sequence of the non-redundant gene set with the KEGG, CAZy and Uniprot databases (the BLASTP alignment parameter sets the expected e-value to 1e-5) to obtain the KEGG, CAZy and Uniprot function corresponding to the gene (Buchfink et al., 2015).

### 2.5. Statistical analysis

One-way analysis of variance was conducted by SPSS 19.0, and all statistical tests considered *p* < 0.05 to be significant. GraphPad Prism 7.0 was used to generate histograms and heat maps. Principal coordinate analysis (PCoA) based on the Bray-Curtis was performed using the “vegan” package of R (version 4.0.2). Co-occurrence network of microbial communities was constructed by calculating the Spearman correlation coefficients (*r* > 0.7, *p* < 0.05) using the relative abundance of OTUs based on the R (Version 4.0.2) package ‘Hmis’. Gephi 0.9.2 and Cytoscape 3.7.1 were used to visualize the network correlations between the microbial communities, the main functional enzymes, and the microorganisms. Based on the R (Version 4.0.2) package “pheatmap,” Pearson’s correlation analysis was performed between the environmental factors and the abundance of microbial community genera. Canoco 5 was used to perform redundancy analyses (RDA) to analyze the correlation between environmental factors and the abundance of microbial community phyla.

## 3. Results

### 3.1. Plant biomass and soil physicochemical properties of 10 different alpine grasslands

The above-ground biomass (AB) and underground biomass (UB) characteristics of different alpine grasslands are summarized in Table 1. AB varied between 85.23–1136.81 g·m^−2^ at the 10 plots. The AB of artificially planted annual oats (G7) was significantly higher than that of other artificial grassland types (*p* < 0.05). The AB of native alpine grasslands was significantly higher than that of degraded grasslands (*p* < 0.05). The UB to a depth of 0–10 cm was 278.69–5623.65 g·m^−2^ at the 10 plots, which is significantly higher than the corresponding AB (except in G7). The UB of alpine swamps was significantly higher than that of alpine meadows, and the UB of alpine meadows was significantly higher than those of alpine steppes, artificial, and degraded grasslands (*p* < 0.05). The degradation of alpine grasslands and the restoration of degraded grasslands to artificial grasslands did not have a significant impact on the UB.

**Table 1:** Characteristics of soil physicochemical properties and vegetation biomass of 10 grassland types.

	G1	G2	G3	G4	G5	G6	G7	G8	G9	G10
MC (%)	87.6 ± 14.29^a^	58.8 ± 3.05^b^	17.53 ± 1.56^de^	25.7 ± 3.74^cd^	22.17 ± 3.4^cd^	22.37 ± 2.06^cd^	28.5 ± 1.91^c^	7.6 ± 0.53^f^	4.63 ± 1.99^f^	12.73 ± 1.17^ef^
pH	5.6 ± 0.1^f^	6.1 ± 0.2^ef^	7.07 ± 0.15^bc^	6.4 ± 0.26^de^	6.9 ± 0.2^cd^	6.7 ± 0.26^cd^	6.8 ± 0.26^cd^	7.8 ± 0.26^a^	7.8 ± 0.6^a^	7.5 ± 0.36^ab^
BD (g/cm^3^)	0.78 ± 0.06^e^	1.08 ± 0.13^d^	1.29 ± 0.06^abc^	1.23 ± 0.03^bcd^	1.25 ± 0.07^bc^	1.35 ± 0.09^abc^	1.2 ± 0.07^cd^	1.35 ± 0.02^abc^	1.41 ± 0.15^a^	1.38 ± 0.12^ab^
TC (g/kg)	165.91 ± 54.08^a^	117.76 ± 23.22^b^	32.44 ± 2.16^c^	33.58 ± 7.54^c^	30.72 ± 6.49^c^	36.55 ± 6.53^c^	32.09 ± 5.31^c^	22.32 ± 5.13^c^	21.16 ± 4.53^c^	28.42 ± 4.17^c^
TOC (g/kg)	161.53 ± 55.18^a^	112.49 ± 20.76^b^	23.17 ± 6.66^c^	27.39 ± 5.43^c^	23.48 ± 3.9^c^	29.69 ± 6.18^c^	22.75 ± 2.98^c^	12.06 ± 4.74^c^	8.57 ± 4.27^c^	19.64 ± 4.83^c^
TN (g/kg)	11.51 ± 2.56^a^	5.98 ± 1.38^b^	3.24 ± 0.76^cd^	3.76 ± 1.03^c^	3.63 ± 1.02^c^	2.71 ± 0.29^cdef^	3.05 ± 0.64^cde^	1.38 ± 0.27^ef^	1.18 ± 0.17^f^	1.73 ± 0.2^def^
SON (mg/kg)	153.32 ± 11.95^a^	66.31 ± 23.62^b^	7.31 ± 0.57^de^	25.63 ± 1.74^c^	18.74 ± 1.56^cd^	15.72 ± 1.96^cde^	12.45 ± 1.88^cde^	2.69 ± 0.78^e^	2.45 ± 0.89^e^	8.76 ± 2.35^de^
NH_4_^+^-N (mg/kg)	50.1 ± 4.64^a^	48.68 ± 16.99^a^	11.44 ± 0.86^def^	27.29 ± 1.9^b^	24.25 ± 2.18^bc^	23.26 ± 2.96^bc^	15.09 ± 2.02^cde^	5.32 ± 0.08^ef^	4.1 ± 1.12^f^	21.21 ± 5.46^bcd^
NO_3_^-^-N (mg/kg)	9.22 ± 1.78^d^	5.31 ± 1.27^e^	13.15 ± 1.04^c^	21.23 ± 1.37^a^	15.71 ± 1.26^bc^	13.53 ± 1.71^c^	17.78 ± 2.16^b^	6.93 ± 1.56^de^	5.12 ± 1.73^e^	15.59 ± 4.04^bc^
TP (g/kg)	1.72 ± 0.29^a^	1.35 ± 0.21^b^	0.75 ± 0.12^de^	1.14 ± 0.11^bc^	0.91 ± 0.12^cd^	0.89 ± 0.16^d^	0.67 ± 0.05^def^	0.43 ± 0.04^f^	0.44 ± 0.08^f^	0.55 ± 0.02^ef^
AP (mg/kg)	11.25 ± 0.88^a^	9.71 ± 0.99^a^	5.39 ± 0.83^c^	7.68 ± 1.75^b^	6.21 ± 1.52^bc^	6.33 ± 0.73^bc^	6.19 ± 1.2^bc^	4.73 ± 0.35^c^	4.89 ± 0.44^c^	5.67 ± 0.73^c^
TK (g/kg)	8.23 ± 1.42^c^	13.78 ± 2.24^ab^	15.58 ± 1.95^ab^	13.59 ± 1.94^b^	17.25 ± 1.75^a^	16.74 ± 2.1^ab^	14.91 ± 2.33^ab^	15.31 ± 1.32^ab^	14.55 ± 1.66^ab^	16.37 ± 3.34^ab^
AK (mg/kg)	101.15 ± 13.75^e^	169.62 ± 40.67^d^	162.24 ± 20.37^d^	177.14 ± 16.77^cd^	216.32 ± 32.88^bc^	273.52 ± 33.61^a^	152.68 ± 20.28^d^	167.89 ± 3.77^d^	140.26 ± 7.95^de^	227.49 ± 33.25^b^
AB (g·m^-2^)	581.11 ± 179.79^b^	438.49 ± 90.86^bc^	85.23 ± 35.52^e^	533.54 ± 61.02^b^	315.32 ± 25.77^cd^	355.94 ± 120.81^cd^	1136.81 ± 208.32^a^	213.16 ± 7.46^de^	126.31 ± 10.14^e^	475.72 ± 55.6^bc^
UB (g·m^-2^)	5623.65 ± 1149.33^a^	3115.17 ± 829.62^b^	278.69 ± 128.67^c^	1145.67 ± 375.44^c^	887.81 ± 238.92^c^	1116.23 ± 478.76^c^	584.23 ± 112.2^c^	816.17 ± 184.74^c^	531.31 ± 254.34^c^	582.26 ± 250.54^c^

MC, moisture content; BD, bulk density; TC, total carbon; TOC, total organic carbon; TN, total nitrogen; SON, soluble organic nitrogen; NH_4_^+^-N, ammonia nitrogen; NO_3_^—^N, nitrate nitrogen; TP, total phosphorous; AP, available phosphorous; TK, total potassium; AK, available potassium; AB, aboveground biomass; UB, underground biomass. In a list, if a minuscule alphabet is same, the divergence is not significant; on the contrary, divergence is significant at 0.05 level.

The quantitative characteristics of soil physicochemical properties of the different alpine grasslands are summarized in Table 1. The MC, TC, TOC, TN, SON, NH_4_^+^-N, TP, and AP in alpine swamps and meadows were significantly higher than those in alpine steppes (*p* < 0.05), and lower in degraded grasslands than in native grasslands and artificially restored grasslands. In contrast, pH and BD in alpine swamp and meadow were significantly lower than in alpine steppe, and increased with the degradation and artificial restoration of grasslands. There was a significant difference in MC between alpine swamp (87.6%), alpine meadow (58.8%) and alpine steppe (7.6%) (*p* < 0.05). The average MC of degraded alpine meadow and associated artificial grasslands was about 23.25%, while the average MC of alpine steppe, degraded steppes and artificial grasslands built on alpine steppes was only 7%. The TC contents of alpine swamp, alpine meadow and alpine steppe were 165.9 g kg^−1^, 117.7 g kg^−1^, and 22.3 g kg^−1^, respectively, and the TN contents were 11.5, 6.0, and 1.4 g kg^−1^, respectively. Significant differences among the three types of native grassland were observed (*p* < 0.05). Degradation of alpine meadow led to a significant decrease in soil TC and TN content, while degradation of alpine steppe and vegetation restoration had no significant effect. It is noteworthy that the NO_3_^−^-N content of artificial grasslands was significantly higher than that of natural grasslands.

### 3.2. Soil microbial characteristics of different alpine grasslands

Thirty soil samples from 0 to 10 cm depth at 10 sites were sequenced for 16S and ITS amplicon diversity, and 2,028,662 16S rDNA valid sequences and 3,496,921 ITS rDNA valid sequences were obtained. OTU clustering was performed based on 97% similarity, with 8,820 16S OTUs and 10,582 ITS OTUs obtained. The rarefaction curves of 10 types of alpine grassland soil samples all tended to be flat (Supplementary Figures S2a,b), and the coverage values were all over 0.95 (Supplementary Figures S2c,d), indicating a near-complete sampling of microorganisms. Alpha-diversity indexes included the Shannon index and Chao1, which, respectively, reflect the uniformity and richness of microbial diversity among different samples (Supplementary Figures S2e–h). The alpha-diversity index of bacteria was not significantly different among the 10 sites (*p* > 0.05), while the alpha-diversity index of fungi did differ significantly (*p* < 0.05). This showed that the uniformity and richness of bacterial diversity were not affected by grassland type, while fungal diversity increases in alpine meadow to varying degrees with soil degradation and artificial restoration, but not in alpine steppe.

**Figure 1:**
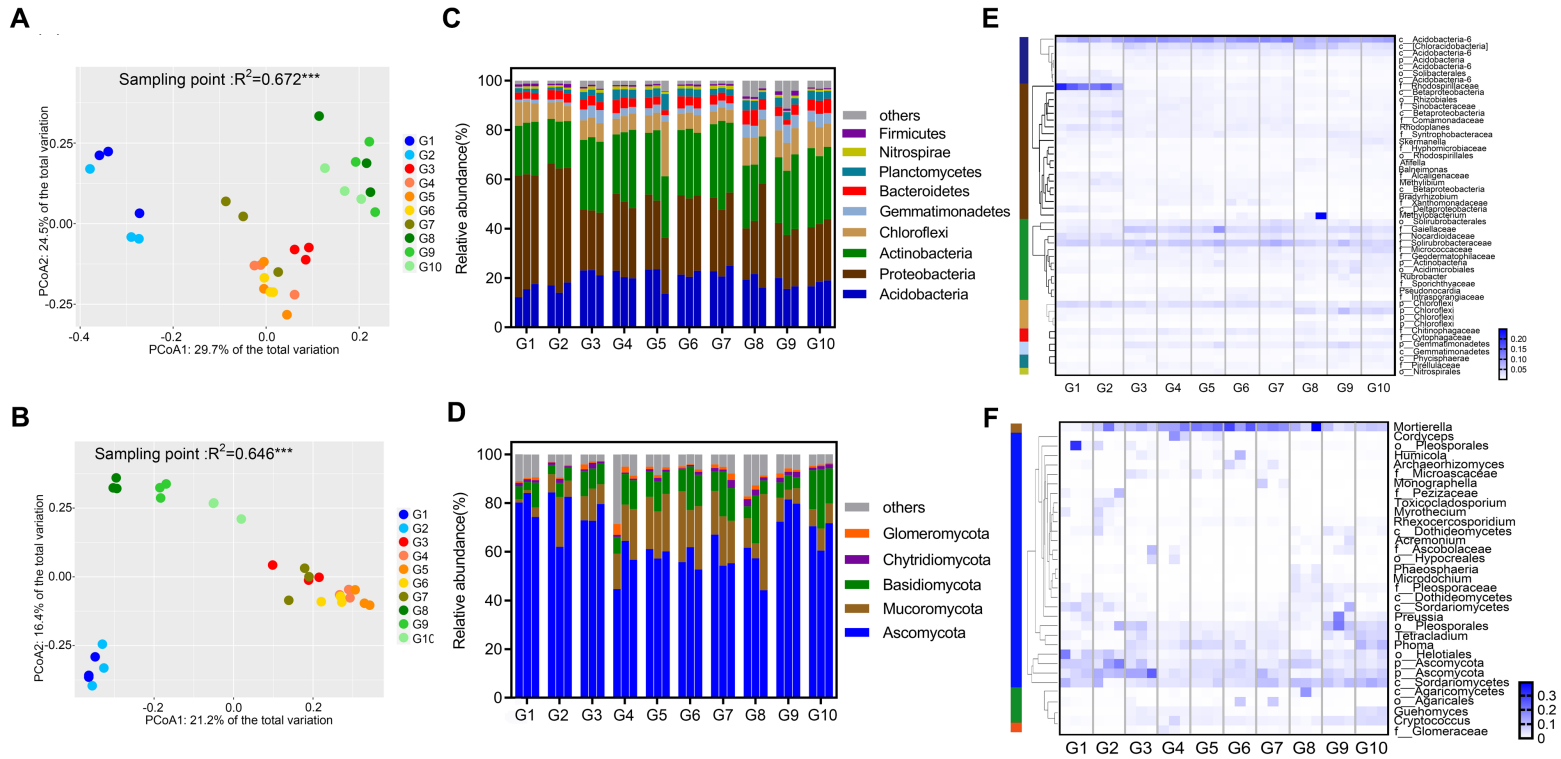
Composition of microbial communities in 10 grassland types. PCoA plots of samples for the bacterial community **(A)** and fungal community **(B)** based on Bray–Curtis distances; Composition of bacteria **(C)** and fungal **(D)** at the phylum level; Heat map showing the genera of bacterial **(E)** and fungal **(F)** communities with relative abundances >1% present in more than half of the sample.

Based on Bray–Curtis distance measurements, PCoA showed significant differences in the community composition of fungi (PERMANOVA; *p* < 0.001, *R*^2^ = 0.676) and bacteria (PERMANOVA; *p* < 0.001, *R*^2^ = 0.672) at the 10 sites (Figures 1A,B). The microbial communities of sites G1–G2, G3–G7, and G8–G10 were all tightly clustered, indicating that the microbial community composition of alpine swamps and alpine meadows were relatively consistent, but were inconsistent on alpine steppes. Further PERMANOVA test on 10 grassland types grouped according to Supplementary Table S2 showed that “land types,” “vegetation types,” and “degradation degree” all had highly significant effects on microbial community composition (Supplementary Figure S3).

### 3.3. Soil microbial community composition of different alpine grasslands

#### 3.3.1. Community composition of bacteria

The relative abundance of soil bacteria at the phylum level in different grasslands on the Tibetan Plateau is shown in Figure 1C. The main soil bacterial phyla in the different grasslands were Actinobacteria, Proteobacteria, and Acidobacteria, which collectively accounted for more than 70% of the total. Among them, the average relative abundance (47.69%) of Proteobacteria in alpine swamps and meadows (G1 and G2) was higher than that in the other sites. However, the relative abundance of the phylum Bacteroidetes, which are Gram-negative bacteria, was low at all 10 sites, with an average relative abundance of 3.97%. Actinobacteria, the second most abundant bacterial phylum, had a higher relative abundance in steppes and artificial grasslands, and a lower relative abundance in alpine swamps and alpine meadows (G1 and G2). The relative abundance of the phylum Firmicutes, which are Gram-positive bacteria, was lower at the 10 sites, with an average relative abundance of only 0.67%. The relative abundances of Acidobacteria and Chloroflexi were approximately the same at the 10 sites, with an average of 19.43 and 8.56%, respectively. The average relative abundance of Gemmatimonadetes was low (1.19%) in alpine swamps and alpine meadows (G1, G2). It was more abundant (2.71%) in artificial grasslands (G4–G7), and in degraded meadows and grasslands its relative abundance was 5.02%.

The differences in soil microbial community composition in different alpine grasslands were then analyzed in more detail by looking at clusters of bacterial genera that appeared with relative abundances of more than 1% in at least half of the samples (Figure 1E). Among these genera, 20 belonged to the Proteobacteria, 12 to the Actinobacteria, seven to the Acidobacteria, and four to the Chloroflexi. The most detected bacterial genera and the more abundant genera showed similar distribution characteristics at the 10 types of alpine grassland sampled. It is worth noting that the relative abundances of Rhodospirillaceae (Proteobacteria) (12.10%) found in alpine swamps and alpine meadows (G1 and G2) were significantly higher than at the other sites. The number of species and abundance of Actinobacteria in degraded and artificial grasslands (G3–G7, G9–G10) were higher than in native alpine grasslands (G1, G2, G8), and higher in alpine steppes (G8) than in alpine swamps and alpine meadows (G1, G2), in which the common genera of Gaiellaceae (3.33%) and Solirubrobacterales (4.77%) were the dominant representatives. The relative abundance of p_Gemmatimonadetes (1.71%) was consistent with the change trend of Actinobacteria. The dominant bacterial genera shared by the 10 sites are two unknown genera of Acidobacteria, c_Acidobacteria-6 (8.86%) and c_ Chloracidobacteria (3.25%), and an unknown genus of Chloroflexi, p_Chloroflexi (2.64%). The relative abundance of unannotated species at the genus level reached 68.33%, indicating that there are still a large number of unknown bacteria to be discovered in the soils of the Tibetan Plateau.

#### 3.3.2. Community composition of fungi

The taxonomic analysis of the ITS rRNA sequences revealed a total of five phyla, with Ascomycota, Mucoromycota, and Basidiomycota being the most abundant, collectively averaging more than 90% of all sequences (Figure 1D). Ascomycota were the most abundant phylum, with an average relative abundance of more than 75% in alpine swamps and alpine meadows (G1–G3) and degraded alpine steppes (G9), while its relative abundance in artificial grasslands was lower. The relative abundance and change trend of Mucoromycota and Basidiomycota at the 10 sites were similar, and their relative abundance in artificial grasslands (G4–G7 and G10) was higher than in native grasslands and degraded grasslands (G1–G3 and G8–G9), the opposite trend in change to Ascomycota. There were also a small number of Chytridiomycota and Glomeromycota, with an average relative abundance of 1.37 and 1.16%, respectively.

The fungal genera with a relative abundance of more than 0.5% appearing in at least half of the samples were clustered as shown in Figure 1F. Among them, 27 genera belonged to the Ascomycota, one belonged to the Mucoromycota, four belonged to the Basidiomycota, and one belonged to Glomeromycota, which is consistent with the data at the phylum level. The genera with the most detected fungal species and the most abundant genera had similar distribution characteristics at the 10 sites. There were many species of Ascomycota, among which c_Sordariomycetes, p_Ascomycota, o_Helotiales, Tetracladium, and Phoma were common and relatively abundant fungal genera in all 10 sites. The most representative fungal genus was Mortierella (Mucoromycota). The relative abundance of unannotated species at the genus level reached 39.52%, indicating that there are also more unknown fungal genera in the soils of the Tibetan Plateau.

### 3.4. Metagenomic characteristics of soil microorganisms

Metagenomic sequencing was performed on the 10 types of alpine grassland soil sample, with an average of 11.75 million reads per sample. From these metagenomes, 11,299,217 non-redundant catalog genes were identified, with an average length of 416.05 bp. The results showed that as the level of functional annotation increased, the average percentage of unannotated sequences increased, from 5.93% at level 2 to 49.20% at level 3, which is consistent with the diversity data. This suggested that there are many original sequences in the alpine grasslands of the Three-River Source Region of the Tibetan Plateau, and that there are a large number of related genes with unknown functions. The functions annotated under KEGG level 1 for the 10 types of grassland included: metabolism (71.91%), genetic information processing (8.84%), environmental information processing (6.92%), cellular processes (5.52%), human diseases (4.08%), and organic systems (2.74%) (Supplementary Table S3). Supplementary Figure S4 showed the relatively rich functional pathways (>1%) at level 3: carbon metabolism, amino acid biosynthesis, ABC transporter, purine metabolism, quorum sensing, and pyrimidine metabolism, which were more represented than other pathways. Among them, alpine swamps (G1) and alpine meadow (G2) were characterized by higher share of ABC transporters, two-component system, pyruvate metabolism, glycolysis, starch and sucrose metabolisms compared to all other sites.

#### 3.4.1. Genome-centric view of polysaccharide degradation

**Figure 2:**
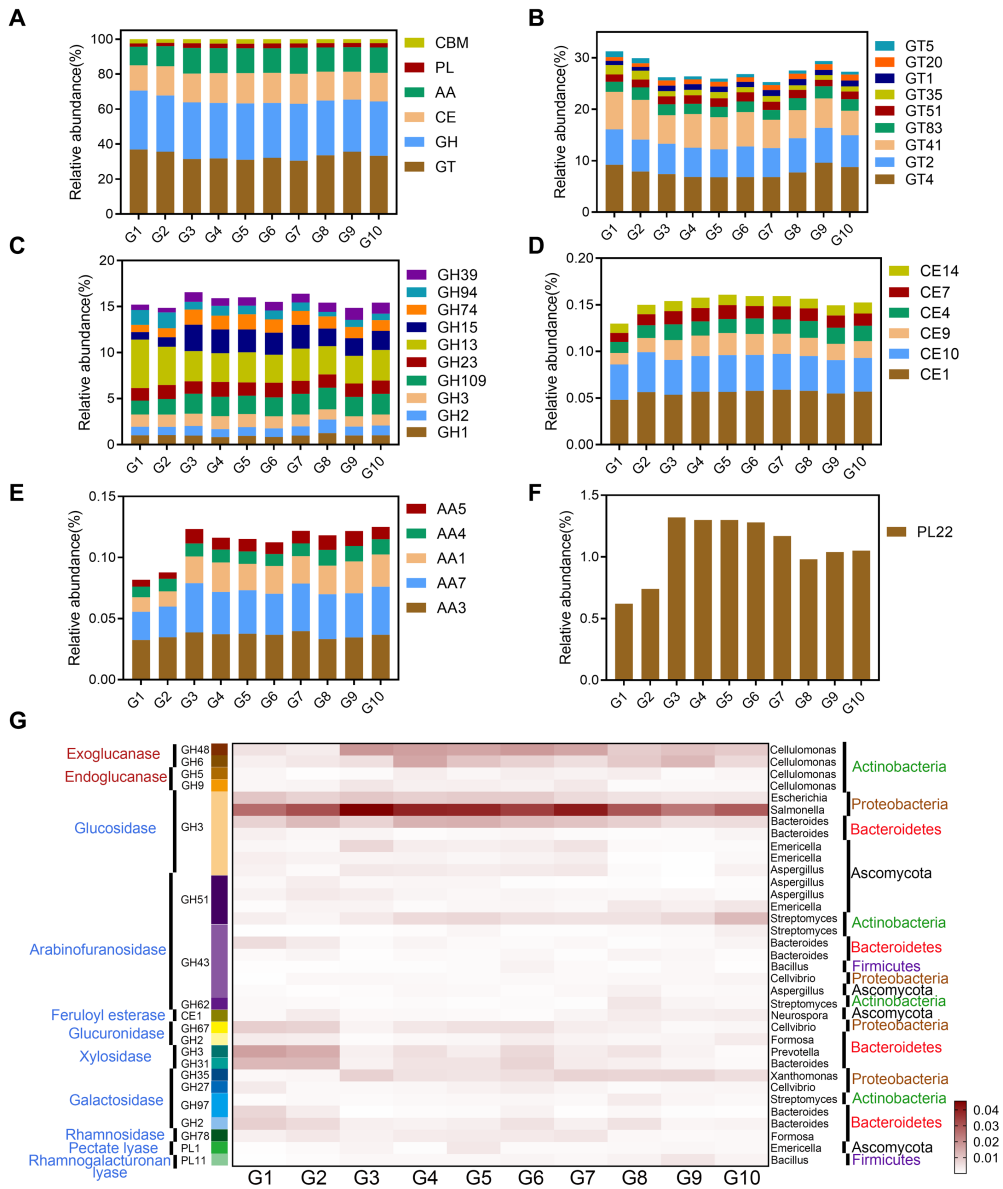
Soil organic carbon degrading enzymes and functional microorganisms in samples G1–G10. **(A–F)** The types and relative contents of CAZymes. **(G)** The heat map of CAZymes with relative abundance >0.002%, and the corresponding microbial classifications of the enzymes.

The results of the metagenomic data analysis suggested that the most abundant enzyme categories in the CAZymes database^4^ were the glycosyltransferases (GTs), and most GTs belonged to GT2 (6.13%) and GT4 (7.78%) (Figures 2A,B). The second most abundant CAZymes were the glycoside hydrolase (GH) family, including amylases of GH13 and GH15 families and (hemi)cellulose degrading enzymes of GH74 and GH94 families (Figure 2C). The carbohydrate esterase (CE) family was dominated by CE1, CE4, CE7 acetyl xylan esterase, and CE10 aryl esterase (Figure 2D). The auxiliary activity enzymes (AA) and polysaccharide lyase (PL) families mainly oxidize refractory lignin and pectin (Figures 2E,F). The relative amounts of the GT family in alpine swamps and alpine meadows (G1 and G2) was higher than in other grasslands, while the relative amounts of the GH, CE, AA, and PL families were lower than in other grasslands.

As shown in Figure 2G, CAZymes related to glycosidic bond degradation were common among the main plant cell wall polysaccharide components in the 10 types of grassland. The results showed that cellulose degrading enzymes include main-chain degrading enzymes and oligosaccharide degrading enzymes, while xylan, xyloglucan, galactomannan, and pectin degrading enzymes were mainly side-chain degrading enzymes (Supplementary Figure S5). Correlation analysis between CAZymes and the microorganisms found suggested that the main enzymes degrading starch and lignocellulose were mainly secreted by bacteria, especially Actinobacteria, and that the contribution of fungi was relatively small (Supplementary Figure S5). Amylases are mainly secreted by *Bacillus* (Firmicutes) (Supplementary Figure S6a). Hemicellulase and pectin degrading enzymes are mainly secreted by Streptomyces (Actinobacteria) and Bacteroides (Bacteroidetes) (Supplementary Figures S6b,d). Cellulase is mainly secreted by Cellulomonas (Actinobacteria). Chitinase is mainly secreted by *Bacillus* and *Mycobacterium*. Microorganisms with specific functions in the soils of the Tibetan Plateau had fewer polysaccharide degrading enzymes, indicating that their metabolic capacity was specific, which may be closely related to the low temperatures, and low carbon and nitrogen availability of the plateau habitat.

#### 3.4.2. Genome-centric view of nitrogen processing

**Figure 3:**
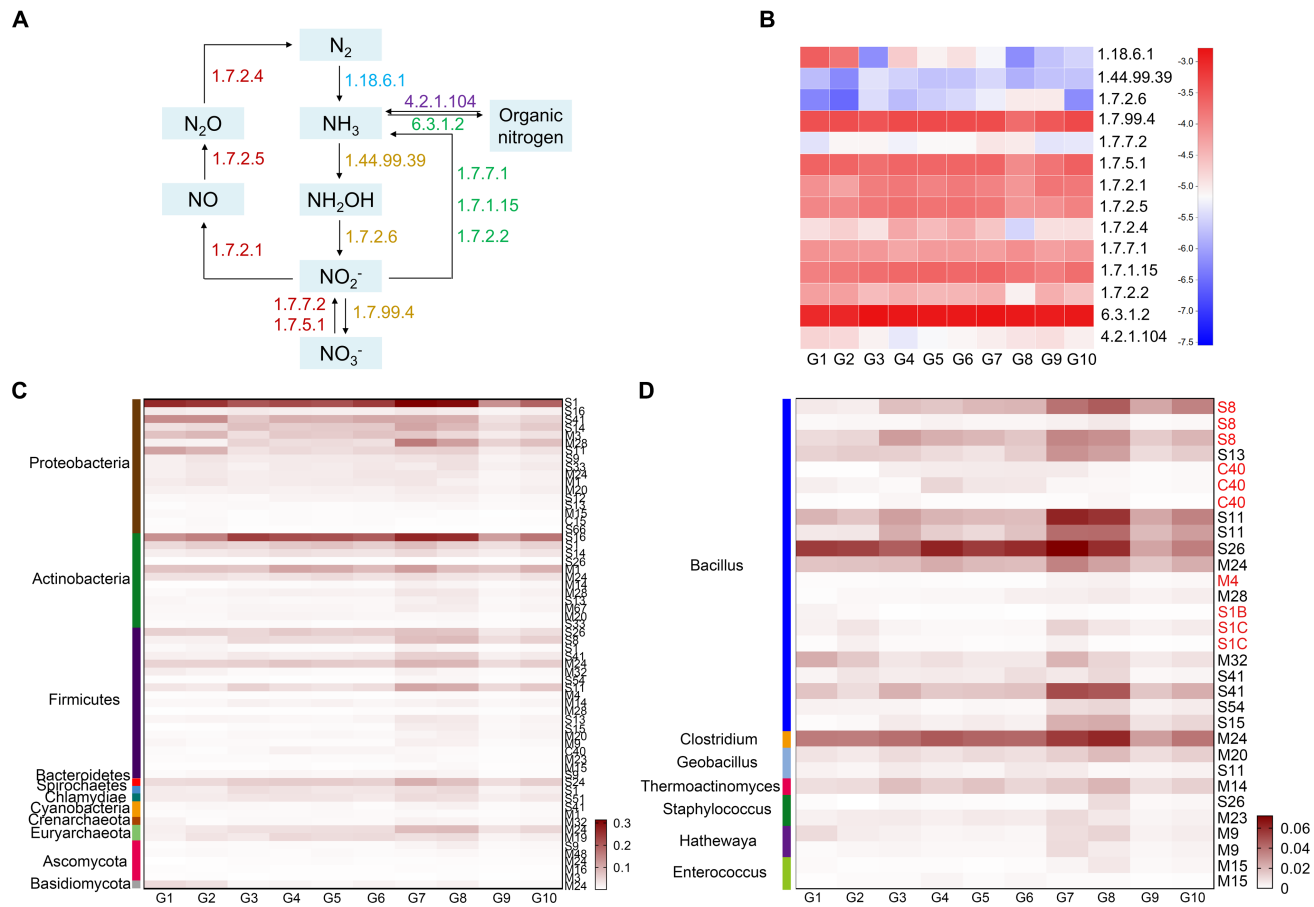
Functional enzymes and key microorganisms in soil nitrogen cycling in the G1–G10 samples. **(A)** Biogeochemical nitrogen cycling revealed by annotating Illumina sequencing datasets. The numbers represent the EC numbers for each enzyme. **(B)** Heat map representing the relative abundance of the enzymes. **(C)** Heat map showing protease families with relative abundance >0.02% in the 10 grassland types. **(D)** Heat map showing protease families from different genera of Firmicutes with relative abundance >0.02%.

Nitrogen is an essential component of living organisms, and is also the main element limiting growth or metabolism of living organisms. The sequencing data were annotated using the KEGG database, which revealed the nitrogen metabolism pathways in the 10 types of grassland (Figures 3A,B). The pathways included: nitrogen fixation (N_2_ → NH_3_), nitrification (NH_3_ → NH_2_OH → NO_2_^−^ → NO_3_^−^), denitrification (NO_3_^−^ → NO_2_^−^ → NO→N_2_O → N_2_), assimilation (NO_3_^−^ → NO_2_^−^ → NH_3_ → Organic nitrogen), and ammonization (Organic nitrogen→NH_3_). Among them, assimilation and denitrification play leading roles. The soil microbial nitrogen fixation capacity (EC 1.18.6.1) of alpine swamps and alpine meadows (G1, G2) were higher than in other grasslands, while nitrification (EC 1.14.99.39, EC 1.7.2.6) was lower than in other grasslands, which is consistent with the fact that alpine swamp and alpine meadow soils are high in ammonium nitrogen and low in nitrate nitrogen (Table 1). In artificial grasslands (G4–G7), the relative abundance of nitrous-oxide reductase (EC 1.7.2.4) for denitrification was high, probably attributable to the reduction of total nitrogen after meadow degradation.

Protein hydrolysis is an important process in the nitrogen cycle and is considered the rate-limiting step in the process of protein mineralization. The relative abundance of proteases in artificially restored grasslands and native grasslands was higher than in native meadows and degraded steppes. Proteobacteria secreted the most protease, with a relative abundance of 0.7226%, followed by the protease secreted by Actinobacteria and Firmicutes, with relative abundances of 0.4834 and 0.3617%, respectively (Figure 3C). Firmicutes secreted the most diverse proteases, including relatively abundant endopeptidases, such as S1, S8, M4, and C40, and exopeptidases such as M24, S13, S11, and S26, which synergistically degrade macromolecular organic nitrogen. These protease families were mainly derived from *Bacillus* and *Clostridium* (Figure 3D).

### 3.5. Association analysis between functional enzyme systems and microbiomes

**Figure 4:**
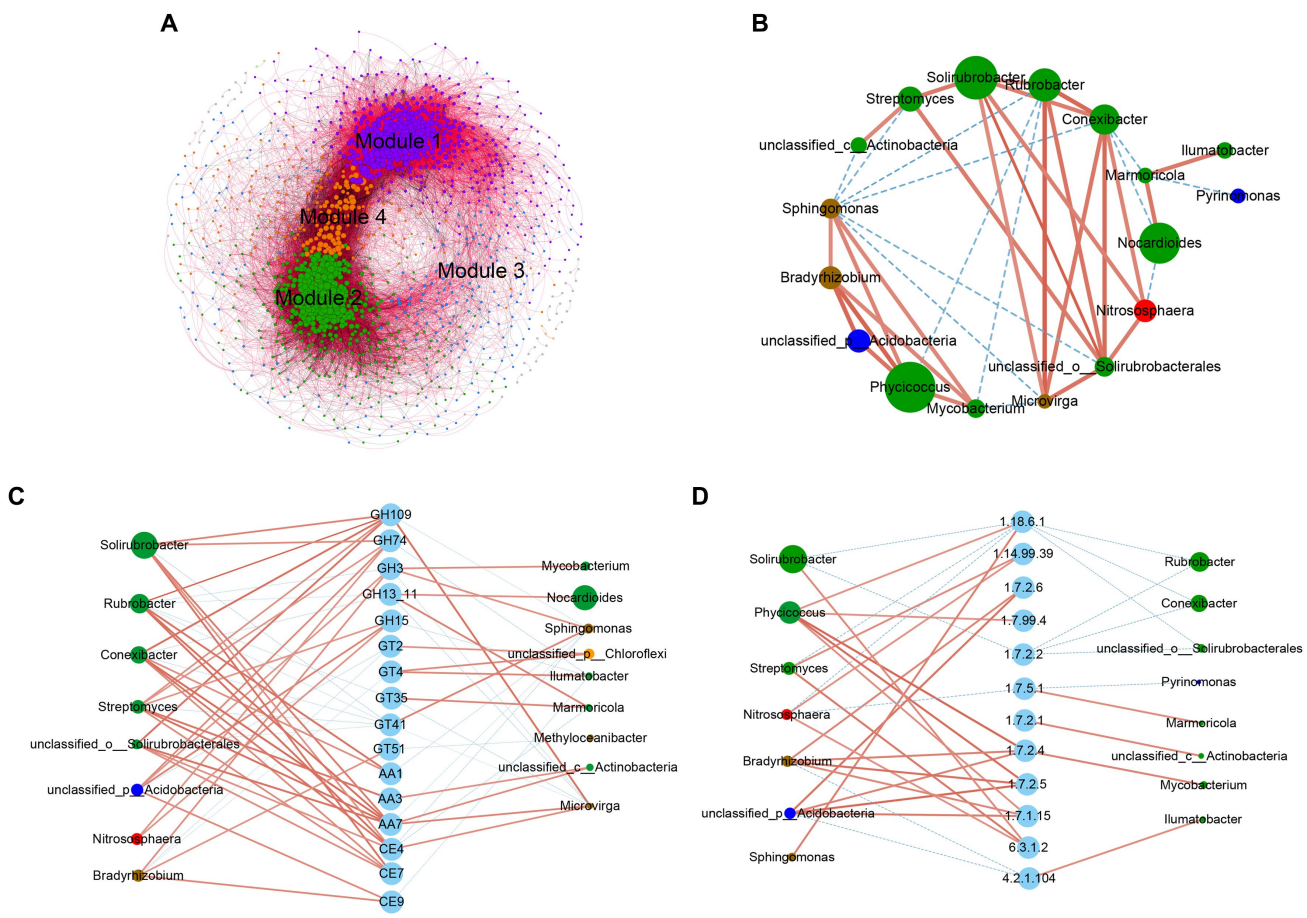
Co-occurrence network analysis based on metagenomic information. **(A)** Network analysis of the associations among bacterial OTUs and fungal OTUs. Nodes represent OTUs and edges represent a significantly strong relationship (*r* > 0.7, *p* < 0.05) between two connected nodes. The size of each node was proportional to the number of edges. The color of each node depends on the module to which it belongs. Purple nodes belong to Module 1, accounting for 35.11% of the total layout, green nodes belong to Module 2, accounting for 31.44%, blue nodes belong to Module 3, accounting for 15.55%, orange nodes belong to Module 4, accounting for 14.17, and the other colors (3.73%) were distributed scattered. The red edges represent two connected nodes with positive correlation and the black edges represent two connected nodes with negative correlation. Details of the nodes clustered in each module and the correlation between the nodes were shown in Supplementary Table S7. The co-occurrences of **(B)** the top 20 dominant genera with **(C)** the top 20 dominant glycoside hydrolases and **(D)** the nitrogen metabolism-related enzymes. The connections between nodes indicate a strong and significant correlation (Spearman’s correlation coefficient *ρ* > 0.5, *p* < 0.05). Node size was weighted according to the degree.

To further determine the potential interactions between microbial communities of the 10 grassland types on the Tibetan Plateau, we constructed a co-occurrence network between bacteria and fungi using network analysis and found four ecological clusters strongly co-occurring with each other (modules 1, 2, 3, and 4; Figure 4A). The network topology and node information of the four main modules was shown in Supplementary Tables S4, S7. More positive interactions (73.04%) were detected in the microbial symbiosis networks. In the composition of the edge, positive bacteria-bacteria interactions accounted for the largest proportion (34.71%), followed by positive bacteria-fungi interactions (23.31%), and fungi–fungi interactions (15.01%). Ascomycota, Proteobacteria, Actinobacteria, and Acidobacteria were relatively dominant, collectively accounting for 74.36% of all nodes. There was a generally positive correlation among the relative abundances of the top 20 dominant bacterial genera. There was a generally negative correlation between *Sphingomonas* and Actinobacteria (Figure 4B), which was closely related to the influence of soil moisture content on different bacteria.

A network analysis was performed on the correlation between CAZymes and nitrogen metabolism functional enzymes in the top 20 most relatively abundant bacterial genera and the top 20 dominant bacterial genera. The results suggested that the genera that correlate most strongly with the functional enzymes regulating carbon and nitrogen metabolism mainly belonged to the Actinobacteria. As shown in Figure 4C, *Nocardioides*, and *Marmoricola* were related to the GH13 family of amylases, and that *Streptomyces*, *Nitrososphaera*, and an unclassified_p_Acidobacteria were related to the GH15 family. The GH74, GH109, and GH3 families of lignocellulose-degrading enzymes were significantly correlated with *Solirubrobacter*, *Phycicoccus* and other bacteria in the Actinobacteria. The enzymes of the GH109 family were mainly alpha-*N*-acetylgalactosaminidases (EC 3.2.1.49), which were relatively more abundant in the 10 types of grassland, and were mainly found in five genera of Actinobacteria and in *Microvirga*. The GH74 family of endoglucanases was positively related to *Streptomyces* and *Solirubrobacter*, and these two bacteria were involved in the degradation process of hemicellulose.

Denitrification is the main pathway for nitrate reduction and accelerated soil nitrogen loss. As shown in Figure 4D, nitrous-oxide reductase (EC 1.7.2.4), the enzyme in the last step of the denitrification pathway, was significantly positively correlated with *Bradyrhizobium*, *Mycobacterium*, *Phycicoccus*, and an unclassified_p_Acidobacteria, with greater abundance in G4–G6 than in the other sites. *Sphingomonas* and *Phycicoccus* were significantly positively correlated with nitrogenase content (EC 1.18.6.1).

### 3.6. Response relationship between soil microorganisms, soil physical and chemical factors, and plant biomass in different alpine grasslands

**Figure 5:**
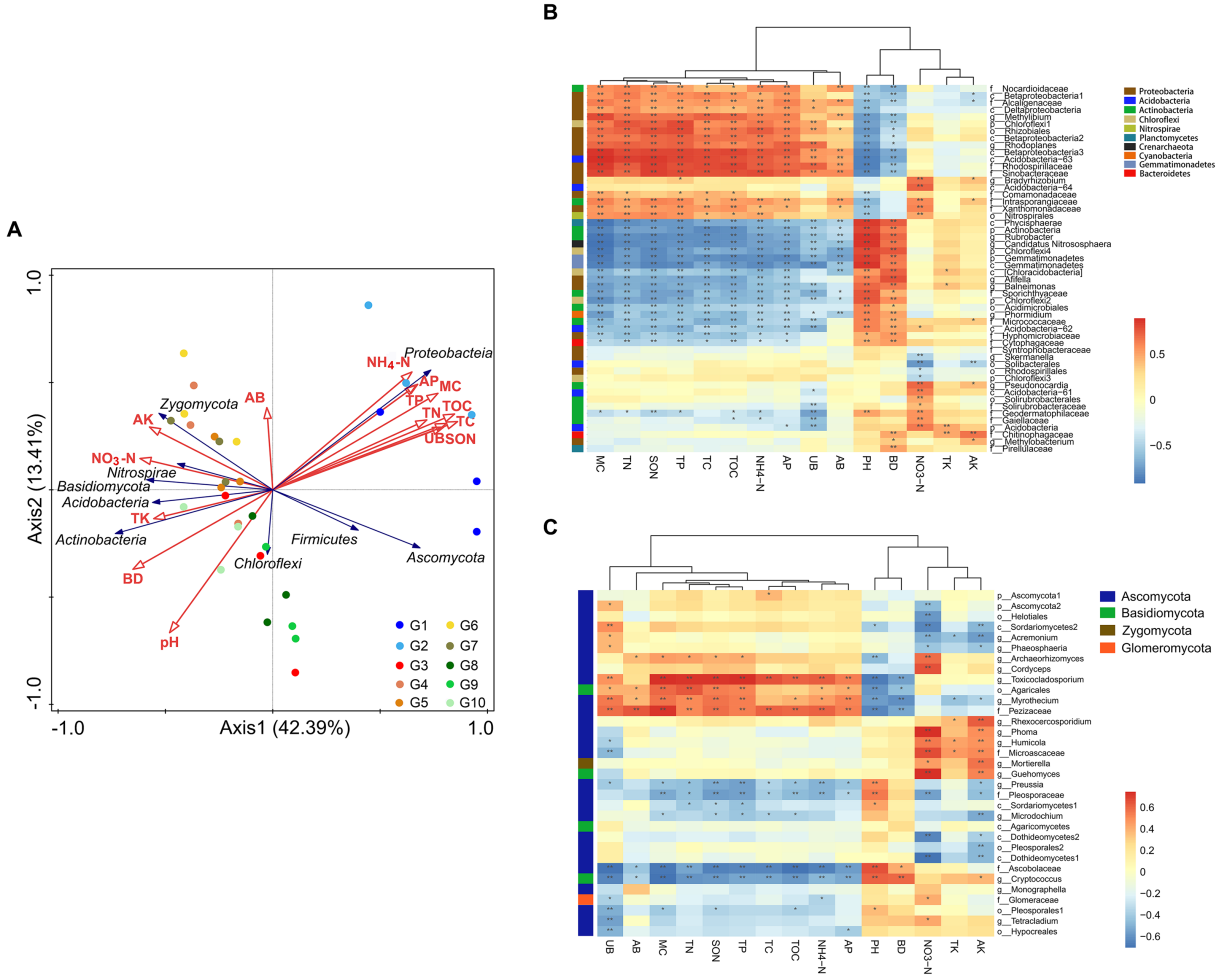
Correlation analysis between microorganisms and soil physicochemical properties and plant biomass in G1–G10 alpine grasslands. **(A)** Redundancy analysis (RDA) of microbial communities with environmental factors and plant biomass. **(B)** Pearson correlation analyses between bacterial communities and environmental factors. **(C)** Pearson’s correlation analyses between fungal communities and environmental factors. The distance between sample points represents the similarities and differences of functional composition between samples. The projected distance from the sample point to the environmental factor represents the degree to which the sample is affected by the environmental factor. The closer the projection line, the more similar the impact of the environmental factor on the two samples. The angle between environmental factors/species represents the positive and negative correlations between environmental factors/species. *p < 0.05; **p < 0.01.

Redundancy analysis was used to explore the correlations between the microbial community compositions and the vegetation and soil properties, which is helpful in understanding the coupling relationships and formation of plant–soil-microbe processes in an ecosystem. As shown in Figure 5A, Proteobacteria were positively correlated with TC, TOC, TN, SON, NH_4_^+^-N, TP, AP, MC, AB, and UB, while most other microorganisms, such as the Actinobacteria, correlated positively with pH, BD, NO_3_^−^-N, TK, and AK. This study also found that the native alpine swamps and alpine meadows (G1 and G2) were situated far away from the other grassland types and were affected by TC, TOC, TN, SON, NH_4_^+^-N, TP, AP, MC, and UB levels to a similar extent. Artificial grasslands (G4–G7) were affected by AB, AK, and NO_3_^−^-N levels, whereas alpine steppes and degraded meadows were affected by pH and BD.

Pearson’s correlation analyses showed that the effects of the 15 soils physical and chemical factors and plant biomass on the bacterial and fungal microbial communities at the genus level could be classified into three categories: (I) TC, TOC, TN, SON, NH_4_^+^-N, TP, AP, MC, AB, UB; (II) pH, BD; and (III) NO_3_^−^-N, TK, AK. As shown in Figure 5B, among the 52 bacterial genera with relative abundance >1%, there were 11 Proteobacteria genera, two Actinobacteria genera, one unidentified Acidobacteria genus, and one unidentified Chloroflexi genus that were significantly positively correlated with environmental factors (I) (*p* < 0.05). Bacteria that were significantly positively correlated with environmental factors (II) included five Actinobacteria genera, three Proteobacteria genera, three unidentified Chloroflexi genera, two unidentified Gemmatimonadetes genera, one Planctomycetes genus, one Acidobacteria genus, one Bacteroidetes genus, *Phormidium* (Cyanobacteria), and Candidatus Nitrososphaera (Archaea). Bacteria that were significantly positively related to NO_3_^−^-N included five Actinobacteria genera, three unidentified Acidobacteria genera, *Bradyrhizobium* (Proteobacteria), and one Nitrospirae genus. The only positive correlation with potassium was f_Chitinophagaceae (Bacteroidetes). Among the 33 fungal genera with relative abundance >0.5%, those with a significant positive correlation (*p* < 0.05) with TC, TOC, TN, SON, NH_4_^+^-N, TP, AP, and MC included *Toxicocladosporium*, and f__Pezizaceae (Figure 5C). Five Ascomycotas (Archaeorhizomyces, *Cordyceps*, *Phoma*, *Humicola*, f__Microascaceae), *Mortierella* (Mucoromycota), and *Guehomyces* (Basidiomycota) were significantly positively correlated with NO_3_^−^-N.

## 4. Discussion

### 4.1. The impact of plant degradation and artificial grassland restoration on plant biomass and soil physicochemical properties

Plants are the main producers in the biosphere, and their aboveground plant residues and underground root systems provide soil microorganisms with organic carbon sources and other essential materials for life (Leff et al., 2018). The dominant species of *Kobresia* spp. (Cyperaceae) in alpine swamps and alpine meadows had higher ground cover than the dominant species of *Stipa purpurea* in alpine steppes, and well-developed root systems distributed in shallow soils, so both AB and UB were higher in alpine swamps and alpine meadows than in alpine steppes. Due to increased rainfall from global warming, there is essentially no degradation of alpine swamp. Alpine meadow degradation significantly reduced plant biomass, while artificial grasslands could only restore AB but not UB. Alpine steppe degradation can restore biomass by artificial reconstruction. Since the biomass is mainly distributed underground, underground biomass is an important component of vegetation carbon accumulation (Fei et al., 2022). The underground part of plants is important for the productivity and ecological functions of grassland ecosystems (mitigating climate warming, contributing to carbon peaking and carbon neutrality, etc.) (Novoplansky, 2019). Underground biomass increased with the years of artificial grassland establishment, which played an important role in preventing grassland degradation, improving soil quality and maintaining sustainable grassland development.

Soil is an important component of terrestrial ecosystems, and provides the material basis for the survival of plants and microorganisms. Its physical and chemical characteristics determine the structural types of plant and microbial communities (Yang and Hu, 2021). In this study, the UB levels followed the same trend as MC, TC, TOC, TN, SON, NH_4_^+^-N, TP, and AP, while the AB had no strong significant correlation with each physicochemical factor (Supplementary Table S5), suggesting that the reduction of plant biomass caused by grassland degradation may be the main reason for decreasing soil C, N, and other nutrients (Wang et al., 2020). The carbon storage of the Tibetan Plateau accounts for about 2.5% of the global soil carbon pool (Wang et al., 2002). The main characteristic of grassland degradation is the decrease of plant biomass, which leads to a decrease in carbon input and loss of soil carbon pool. However, degraded meadows cannot restore soil nutrients and UB through multi-year grassland restoration, whereas degraded steppes can restore soil nutrients and plant biomass through grassland restoration. Therefore, we need to strengthen the ecological protection of alpine meadows to avoid irrecoverable degradation.

### 4.2. The impact of plant degradation and artificial grassland restoration on the composition of soil microbial communities

In this study, the degradation or artificial restoration of grasslands in the Three-River Source Region of the Tibetan Plateau did not change the alpha-diversity of the bacterial communities, which is consistent with previous studies (Zhou et al., 2019). PCoA analysis suggested that the microbial communities of the 10 types of grassland were clustered into three groups, indicating that different grassland types had a greater impact on the composition of microbial communities compared to degradation and restoration. A recent study showed that grassland degradation or artificial restoration changed plant coverage and soil physical and chemical properties, thereby affecting the soil bacterial community structure (Wang H. L. et al., 2021). After meadow degradation, the soil moisture content and the relative abundance of Proteobacteria were significantly decreased, and Proteobacteria grew better in swamp and meadow environments with high moisture contents (Chen et al., 2019). The relative abundance of Actinobacteria increased when meadows were degraded (i.e., suffered decreased moisture content and increased pH) (Li et al., 2021). Actinobacteria abundance can therefore be used as a biomarker to indicate meadow degradation. Degradation and artificial restoration of alpine steppes did not cause changes in Proteobacteria and Actinobacteria, indicating that short-term changes in AB did not significantly affect microbial composition, which may still be closely related to soil organic carbon levels (Liu et al., 2018). It appears that the composition of prokaryotes in soils is more obviously affected by moisture (Castro et al., 2010; Xiao et al., 2020), and that grassland degradation and artificial grassland establishment had an insignificant effect on the abundance of dominant bacteria.

Gemmatimonadetes are typical photoheterotrophic bacteria that can utilize organic carbon for growth and metabolism, and their growth is significant promoted by light (Koblížek et al., 2020). Their relative abundance increased with grassland degradation and was negatively correlated with soil moisture content, indicating their adaptation to dry grassland soils (DeBruyn et al., 2011). The relative abundance of other abundant phyla such as Acidobacteria and Chloroflexi varied less among the 10 types of grassland and may be important in organic matter decomposition and nutrient cycling (Fang et al., 2017; Eichorst et al., 2018).

There are few reports on the composition and structure of soil fungal communities on the Tibetan Plateau. In the present study, the relative abundance of Ascomycota in swamps, meadows, and degraded grasslands was higher than that in artificial and alpine steppes, while the relative abundance of Mucoromycota and Basidiomycota in artificial grasslands was higher than in meadows. This indicated that not only MC had an important effect on fungal community composition (Zhang et al., 2016), human activities such as re-tillage and grazing are also important. The predominance of Ascomycota in all soil samples may be related to their ability to degrade cellulose and hemicellulose (Liers et al., 2006; Shary et al., 2007). The average relative abundance of Basidiomycota and Mucoromycota in artificial grasslands was higher than in grasslands and meadows, and previous studies have reported their close relationship to the degradation of complex lignocelluloses (Lundell et al., 2010; Huhe et al., 2017). At the genus level, the average relative abundance of the unclassified Agaricomycetes (Basidiomycota) (6.18%) in G8 was significantly different from other plots (*p* < 0.05). The native grasslands were suitable for Agaricomycetes fungal growth. Mushroom rings were common in the grassland environments, and can either promote or inhibit the growth of plants (Shantz and Piemeisel, 1917).

### 4.3. Genome-centric view of polysaccharide degradation and nitrogen processing

Typical vegetation types on the Tibetan Plateau (alpine swamps, alpine meadows, and alpine steppes) were studied to explore the carbon and nitrogen metabolic pathways of microbes occurring in degraded and artificial grasslands. In this study, the CAZymes involved in carbon degradation in the organic layer of the permafrost regions of the Tibetan Plateau were mainly secreted by Actinobacteria. However, Woodcroft et al. (2018) used macrotranscriptomic and macroproteomic data to show that the expression of cellulase by Actinobacteria in the thawed permafrost in the Arctic was low, and that the microorganisms encoding cellulase and xylanase mainly belonged to the Acidobacteria, which may be related to arctic environmental factors such as rich carbon and oxygen and lush vegetation. The multi-layer compositional structure of plant cell walls forms a lignocellulose biomass anti-degradation barrier, which is a protective layer against external adverse factors (Zoghlami and Paës, 2019). Therefore, a variety of specific or auxiliary enzymes are needed to degrade the bonds of the various components.

Among CAZymes annotated in the different types of grassland soils on the Tibetan Plateau, those potentially involved in microbial metabolism related to cellulose degradation are more common than those related to hemicellulose degradation. The degradation of cellulose mainly depends on the hydrolysis of GH5, GH6, GH9, GH48, and GH94, indicating that anaerobic microorganisms are mainly involved (Artzi et al., 2015). In this study, *Sphingomonas* (Proteobacteria) was significantly positively correlated with GH94 (Supplementary Table S6). This differed significantly from other aerobic and anaerobic cellulose decomposing microorganisms, whose zymograms are mainly in the GH5, GH7, GH12, and GH45 families (Champreda et al., 2019). In particular, the GH94 family contains cellobiose phosphorylase, which can usually cleave cellobiose into glucose and glucose 1-phosphate (G1P) without consuming ATP in the presence of inorganic phosphate under anaerobic conditions. This could be a major means of energy-saving degradation on the plateau (Kim et al., 2018). GH1, GH2, and GH74 are mainly involved in the degradation of hemicellulose, indicating that the degradation energy is limited and can only complete the degradation of side-chains and oligosaccharides (Arnal et al., 2019). The degradation of lignin is mainly completed by the oxidation of the AA1, AA4, AA7, CE4, and CE7 families of enzymes. Multiple esterase families indicate that on the plateau, plant xylan has undergone various types of acetylation and other modifications (Puchart et al., 2019). The degradation of starches was mainly attributed to amylases in the GH13 and GH15 families, whereas pectin was mainly degraded by PL22 and AA5 families of enzymes. The microorganisms on the Tibetan Plateau mainly oxidatively degrade lignin, and hydrolyze cellulose and hemicellulose side-chains for further degradation. Due to the lack of abundant, efficient degradation enzyme systems, plant litter could not be degraded and transformed efficiently, so that changes in AB did not affect microbial community structure in the short term.

Nitrogen is a limiting nutrient in plateau ecosystems, and so plays a vital role in ecosystem productivity. Nitrogen fixation is the main means by which microorganisms obtain nitrogen resources. *Sphingomonas* and *Phycicoccus* were significantly positively correlated with nitrogenase (1.18.6.1), with *Sphingomonas* being relatively more abundant in meadows with a higher moisture content (2.6%), and *Phycicoccus* having a higher relative abundance (1.7%) in artificially restored meadows. Because nitrogen fixation mostly occurs in an anaerobic environment, meadows with high moisture content and low dissolved oxygen are natural habitats for microorganisms to fix nitrogen efficiently (Zhang et al., 2020). Artificial restoration of grasslands will also increase the abundance of nitrogen-fixing microorganisms, thereby increasing soil nitrogen content. Denitrification is the most important microbial nitrogen-loss pathway in wetland soils (Wang et al., 2019). The relative abundance of nitrous oxide reductase (1.7.2.4) in artificial grasslands was high, which may be one of the factors causing the decrease in TN after meadow degradation. Protein is an important component of soil organic matter (9–16%) and the central pool of organic nitrogen (40%). Proteolysis by protease is the first step in protein mineralization (Greenfield et al., 2020). In this study, Firmicutes could produce a more comprehensive family of proteases to synergistically degrade organic nitrogen, although their abundance in the plateau environment overall was low (<1%) and significantly different from the microbial community structures in crop rhizospheres, compost, and other habitats (Lee et al., 2021; Zhang et al., 2021). This may be a reason for the low protease abundance in the 10 alpine grasslands with little difference. *Bacillus* spp. can secrete many endopeptidases, such as S1, S8, M4, and C40, and exopeptidases such as M24, S13, S11, and S26 to synergistically degrade macromolecular organic nitrogen. A recent study showed that proteases from the genus *Bacillus* stimulated microbial activity, which in turn promotes the mineralization of organic matter and phosphorus (Caballero et al., 2020). Therefore, adding an appropriate number of low temperature *Bacillus* to the plateau environment could promote the conversion of organic nitrogen to available nitrogen.

Available phosphorus also commonly limits the success of ecosystem restoration (Wang H. L. et al., 2021). Organic phosphorus in the plateau soils require mineralization by phosphatase to release available phosphorus for plants. Phosphatases including alkaline phosphatase and acid phosphatase can catalyze the hydrolysis of phospholipids to release phosphate (Hu et al., 2018). Therefore, phosphatase could be a good indicator of the mineralization potential and biological activity of organic phosphorus in the soil. Metagenomic data showed that the average alkaline phosphatase content in G8–G10 (0.17%) was higher than in the other plots (0.14%), which is consistent with the higher ratio of available phosphorus to total phosphorus in G8–G10 (Supplementary Figure S7).

## 5. Conclusion

This study elucidated the distribution patterns of soil nutrient and microbial communities in different alpine grassland types on the Tibetan Plateau and further revealed the functional characteristics of carbon and nitrogen degradation. The results showed that alpine meadow degradation caused irreversible reduction in soil nutrients and significant changes in microbial composition, while steppe degradation can restore soil nutrients and microbes through artificial restoration. With the degradation (i.e., decrease in moisture content) and artificial restoration of alpine meadows, the dominant bacterial shifted from Proteobacteria to Actinobacteria, while the relative abundance of Mucoromycota and Basidiomycota among fungi increased. Temporary degradation of alpine vegetation will not affect the community composition of soil microorganisms due to the lack of comprehensive and effective enzymes for organic carbon and nitrogen degradation by plateau microorganisms. In addition, efficient CAZymes in the genomes of *Solirubrobacter*, *Phycicoccus* and *Rubrobacter* had the potential to degrade plant residues. Nitrogenase (EC 1.18.6.1) secreted by *Sphingomonas* were associated with nitrogen fixation in alpine meadows with high moisture content. *Phycococcus* both fixed nitrogen and release it through the secretion of nitrous oxide reductase (EC 1.7.2.4) involved in denitrification in degraded and artificial meadows. Firmicutes with the comprehensive protease system (S8, S11, S26, and M24) and amylase system (GH13, GH15) were less abundant on the Tibetan Plateau, therefore, the addition of functional bacteria such as low temperature *Bacillus* may promote the conversion of organic nitrogen to available nitrogen. These findings provide a comprehensive understanding of the microbial carbon and nitrogen degradation in the Tibetan Plateau, thereby laying a reasonable foundation for more effective restoration of degraded grasslands.

## Data availability statement

The datasets presented in this study can be found in online repositories. The names of the repository/repositories and accession number(s) can be found at: NCBI Sequence Read Archive (SRA) with submission ID PRJNA952838, PRJNA953334, and PRJNA9529923.

## Author contributions

CY: investigation, methodology, and data curation. HZ: conceptualization, investigation, date analysis, and writing – original draft and review. XZ: conceptualization, supervision, and validation. PL: formal analysis, methodology, and data curation. LW: conceptualization, formal analysis, and writing – review and editing. WW: supervision, conceptualization, funding acquisition, project administration, and writing – review and editing. All authors contributed to the article and approved the submitted version.

## Funding

This work was supported by the 2021 first funds for central government to guide local science and technology development in Qinghai Province (2021ZY002), the Second Tibetan Plateau Scientific Expedition and Research (STEP) Program (2019QZKK0302), The 2020 Joint Research Project of Three-River National Park of the Chinese Academy of Sciences and the People’s Government of Qinghai Province (LHZX-2020-08).

## Conflict of interest

The authors declare that the research was conducted in the absence of any commercial or financial relationships that could be construed as a potential conflict of interest.

## Publisher’s note

All claims expressed in this article are solely those of the authors and do not necessarily represent those of their affiliated organizations, or those of the publisher, the editors and the reviewers. Any product that may be evaluated in this article, or claim that may be made by its manufacturer, is not guaranteed or endorsed by the publisher.

## Footnotes

^1^http://drive5.com/uparse/

^2^http://www.arb-silva.de

^3^http://unite.ut.ee/index.php

^4^http://www.cazy.org/
