# Supplementary material for: A functional metagenomics study of soil carbon and nitrogen degradation networks and limiting factors on the Tibetan plateau
Source: Front Microbiol. 2023 May 5;14:1170806. doi: 10.3389/fmicb.2023.1170806 (PMC10203874; doi:10.3389/fmicb.2023.1170806)
Supplement: Supplementary file 1 [file Data_Sheet_1.docx]

***Supplementary Material***

A functional metagenomics study of soil carbon and nitrogen degradation networks and limiting factors on the Tibetan Plateau

**Chong Yang^1,2†^, Hong Zhang^3†^, Xinquan Zhao^4^, Pan Liu^1^, Lushan Wang^3^, Wenying Wang^2*^**

^1^School of Geography Sciences, Qinghai Normal University, Xining, China

^2^School of Life Sciences, Qinghai Normal University, Xining, China

^3^State Key Laboratory of Microbial Technology, Shandong University, Qingdao, China

^4^Northwest Plateau Institute of Biology, Chinese Academy of Sciences, Xining, China


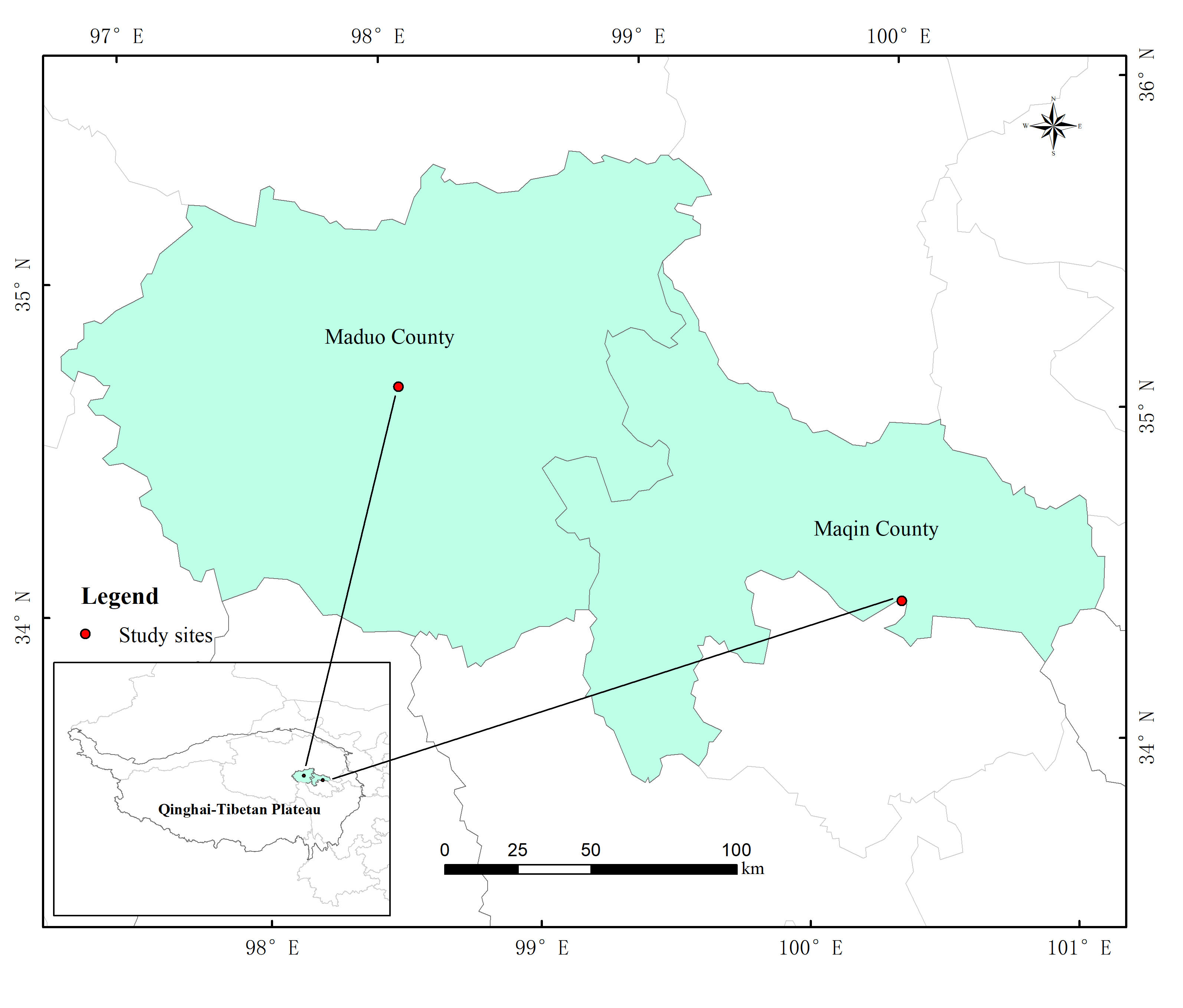


Figure S1. Geographical sampling locations in the study sites

Table S1 Plot details.

| Plot | Site | Grassland type | GPS information | Altitude (m) | Dominant species | Vegetation total cover (%) |
| --- | --- | --- | --- | --- | --- | --- |
| G1 | Maqin | Alpine swamp (winter grazing) | 34°28′0044″N 100°13'2234''E | 3730 | *Kobresia tibetica, Blysmus sinocompressus* | 93 |
| G2 | Maqin | Alpine meadow (winter grazing) | 34°21'4460''N 100°23'1669''E | 3946 | *Kobresia humilis, Kobresia capillifolia* | 94 |
| G3 | Maqin | Degraded alpine meadow (winter grazing) | 34°21'3446''N 100°29'7276''E | 3953 | *Lancea tibetica, Artemisia hedinii* | 24 |
| G4 | Maqin | Artificial restored grassland (6 years + grazing prohibition) | 34°21'3148''N 100°29'6773''E | 3956 | *Elymus nutans, Poa crymophila* | 93 |
| G5 | Maqin | Artificial restored grassland (6 years + winter grazing) | 34°21'3510''N 100°29'6841''E | 3954 | *Elymus nutans, Pedicularis kansuensis* | 91 |
| G6 | Maqin | Artificial restored grassland (16 years + winter grazing) | 34°21'5004''N 100°29'9304''E | 3958 | *Elymus nutans,* *Pedicularis kansuensis* | 92 |
| G7 | Maqin | Artificial oat grassland (1 year + winter grazing) | 34°21'2865''N 100°29'7832''E | 3955 | *Avena sativa* | 90 |
| G8 | Maduo | Alpine steppe (winter grazing) | 34°52'4036''N 98°14'9423''E | 4227 | *Stipa purpurea* | 67 |
| G9 | Maduo | Degraded alpine steppe (winter grazing) | 34°52'3805''N 98°14'9876''E | 4226 | *Potentilla anserina, Leontopodium nanum* | 32 |
| G10 | Maduo | Artificial restored grassland (5 years + winter grazing) | 34°50'7841''N 98°26'5265''E | 4224 | *Elymus nutans* | 82 |


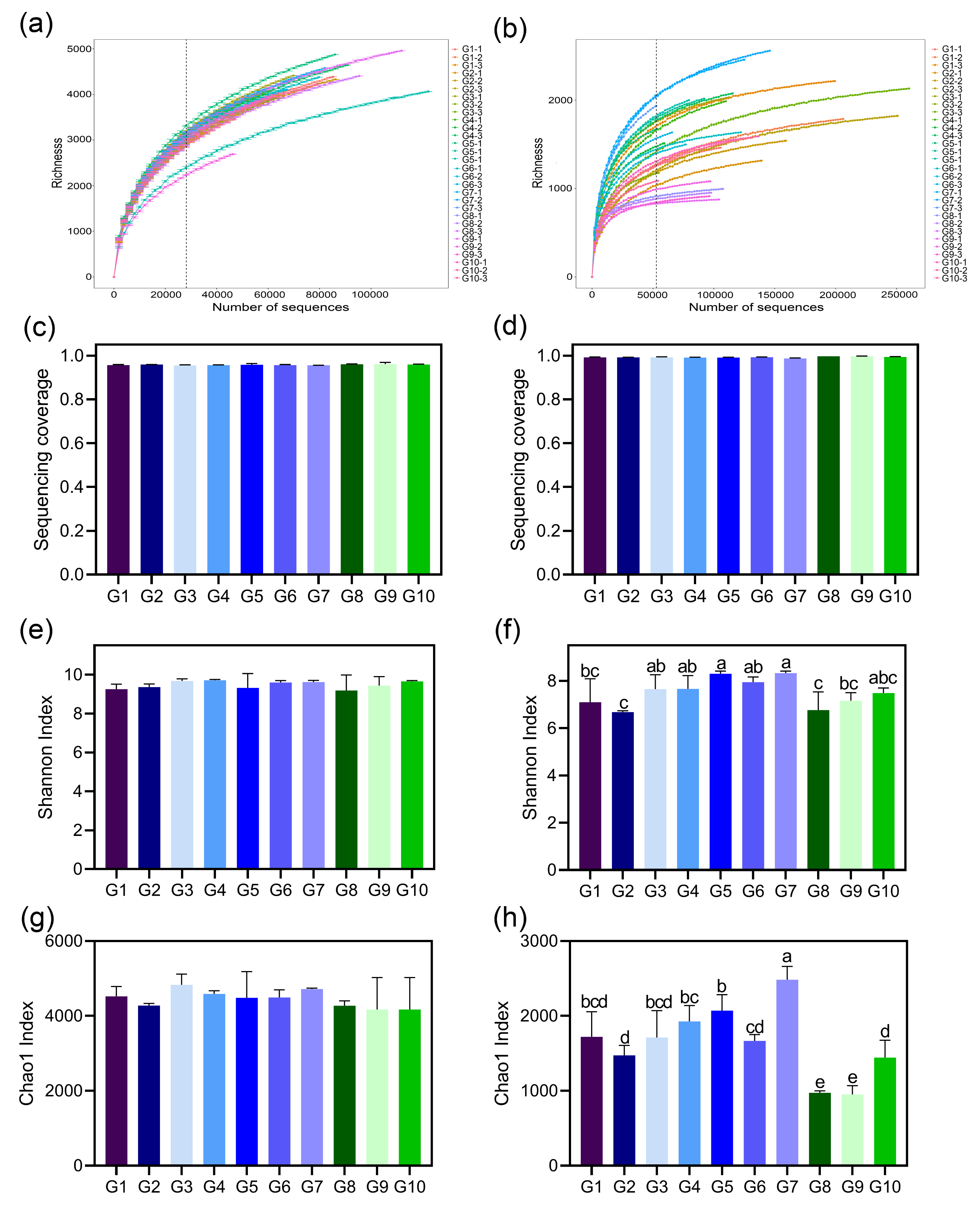


Figure S2. Alpha diversity of 10 alpine grasslands. (a) Bacterial rarefaction curve; (b) Fungal rarefaction curve; (c) Bacterial sequencing coverage; (d) Fungal sequencing coverage; (e) Bacterial Shannon index; (f) Fungal Shannon index; (g) Bacterial Chao1 index richness; (h) Fungal Chao1 index richness.

Table S2 Grouping the samples according to different treatments.

|  | different treatments | group | samples |
| --- | --- | --- | --- |
| land types | Swamp | S1 | G1 |
|  | Meadow | S2 | G2-G7 |
|  | Steppe | S3 | G8-10 |
| vegetation types | Cyperaceae | P1 | G1, G2 |
|  | Scrophulariaceae | P2 | G3, G5 |
|  | Gramineae | P3 | G4, G6-G8, G10 |
|  | Compositae | P4 | G9 |
| degradation degree | Native grassland | D1 | G1, G2, G8 |
|  | Degraded grassland | D2 | G3, G9 |
|  | Artificial grassland | D3 | G4-G7, G10 |


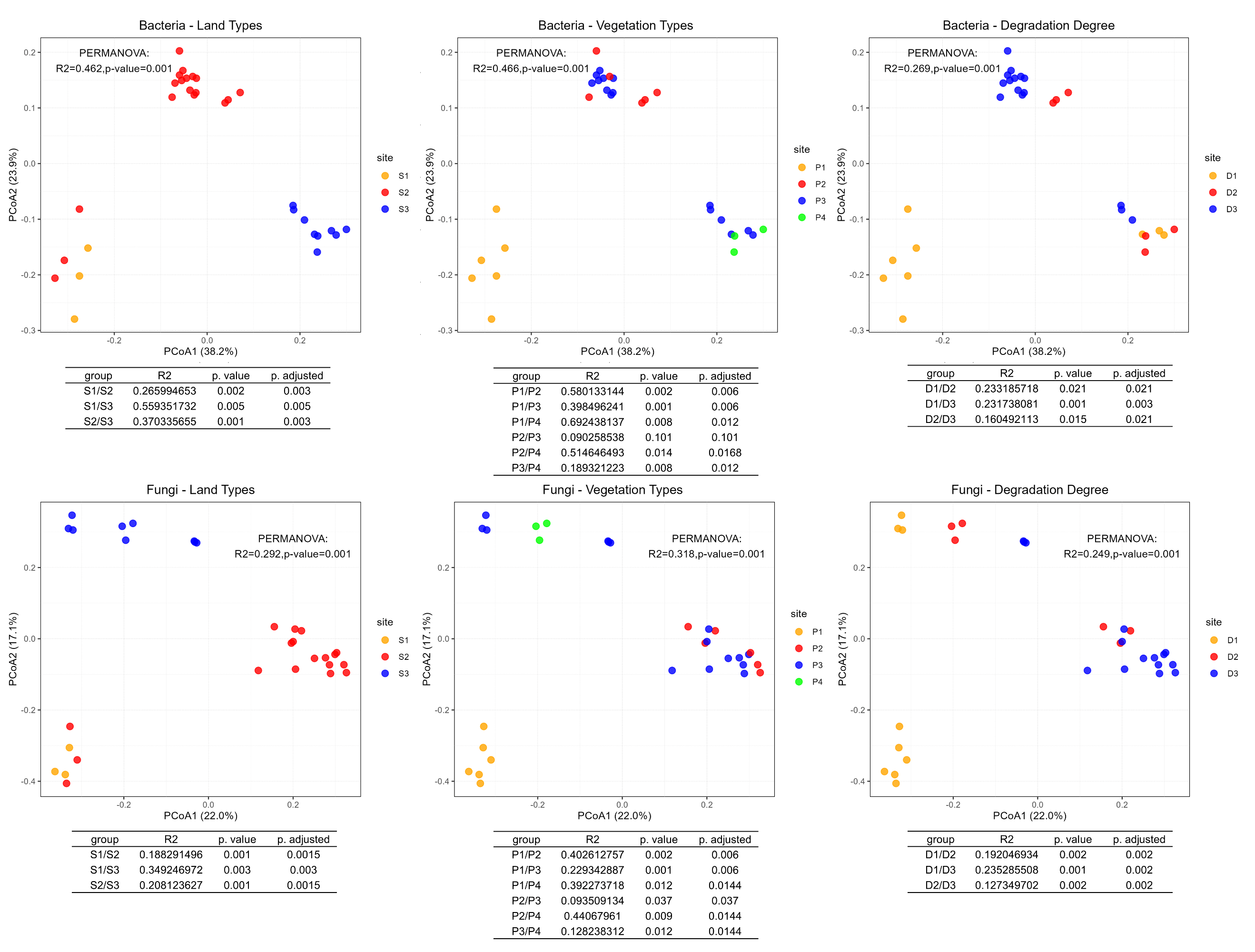


Figure S3. PCoA plots of microbial communities based on different treatments of Table S2 and PERMANOVA tests of the differences in bacterial and fungi community structure based on Bray-Curtis distance.

Table S3 The relative abundance of the total metabolic pathways at level 1 of G1-G10.

|  | Cellular Processes | Environmental Information Processing | Genetic Information Processing | Human Diseases | Metabolism | Organismal Systems |
| --- | --- | --- | --- | --- | --- | --- |
| G1 | 6.28% | 7.79% | 8.41% | 4.43% | 70.13% | 2.97% |
| G2 | 6.30% | 7.84% | 8.15% | 4.40% | 70.42% | 2.88% |
| G3 | 5.29% | 6.51% | 9.03% | 3.97% | 72.44% | 2.77% |
| G4 | 5.42% | 6.90% | 8.88% | 4.13% | 71.91% | 2.76% |
| G5 | 5.46% | 6.86% | 8.79% | 4.08% | 72.01% | 2.80% |
| G6 | 5.50% | 6.96% | 8.81% | 4.23% | 71.76% | 2.73% |
| G7 | 5.34% | 6.72% | 8.93% | 4.08% | 72.12% | 2.80% |
| G8 | 5.17% | 6.46% | 9.21% | 3.87% | 72.65% | 2.63% |
| G9 | 5.25% | 6.58% | 9.19% | 3.81% | 72.66% | 2.51% |
| G10 | 5.17% | 6.53% | 8.97% | 3.76% | 72.98% | 2.59% |


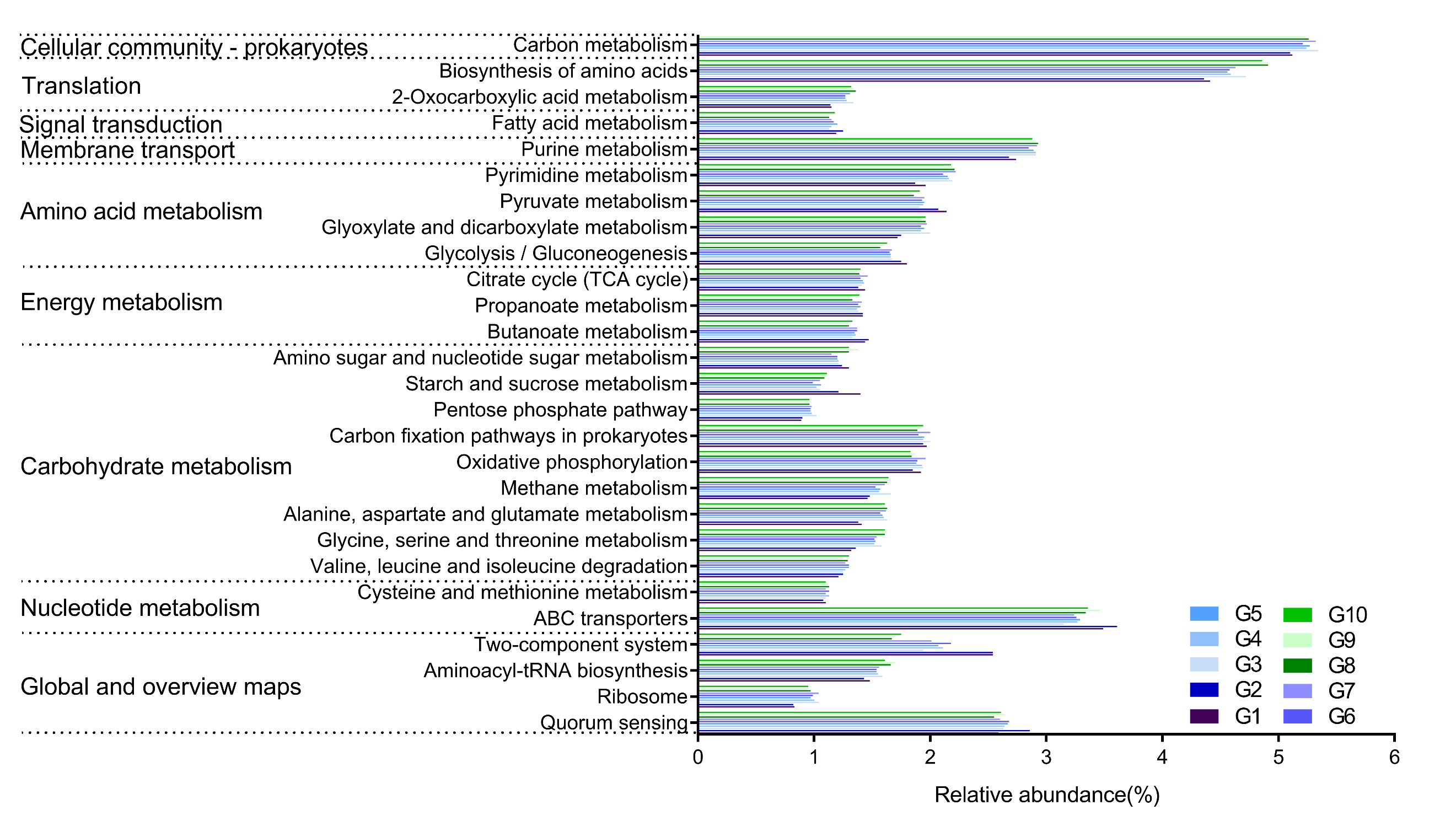


Figure S4. The relative abundance (>1%) of the total metabolic pathways at level 3 (%) of G1-G10.


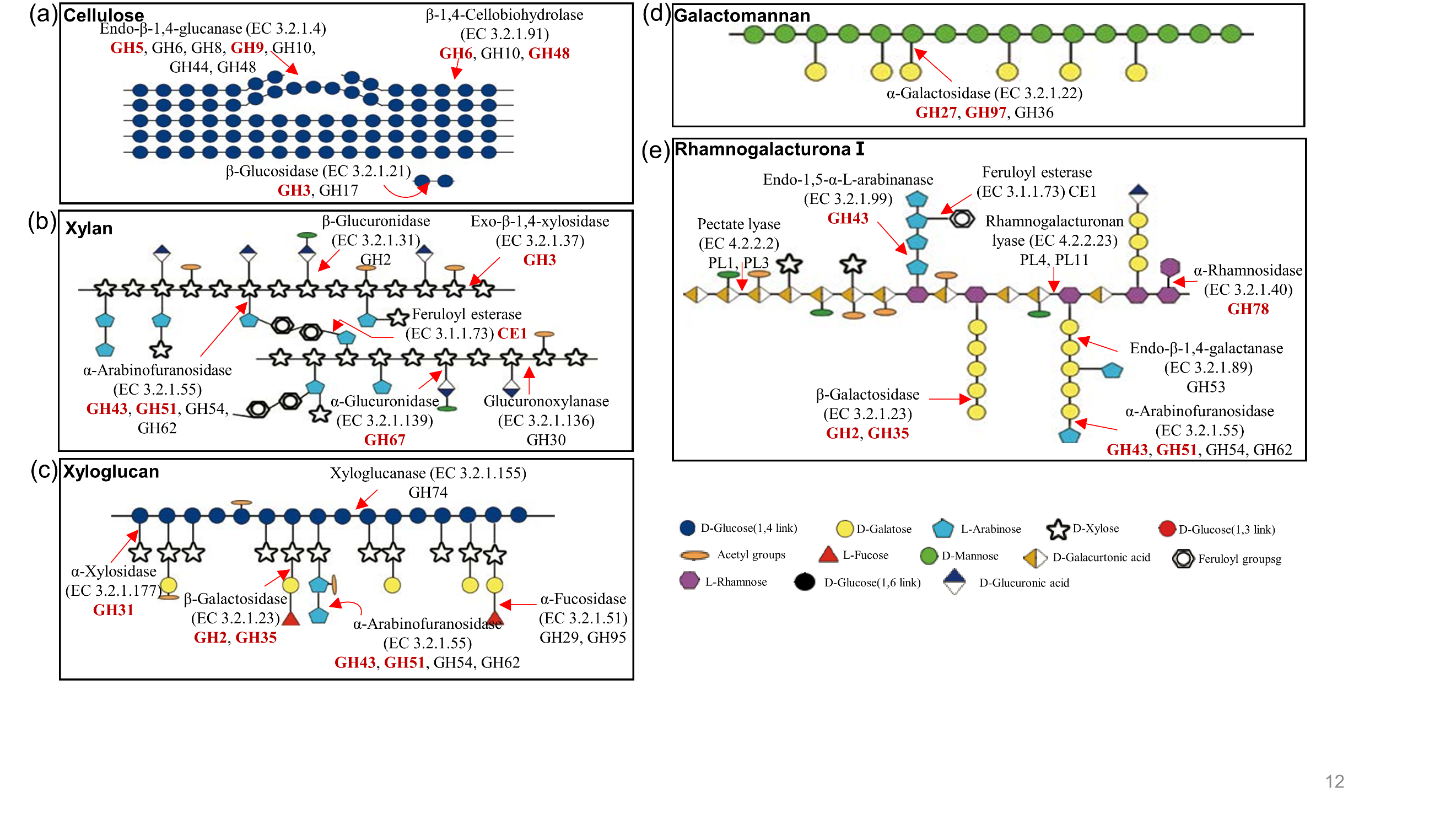


Figure S5. Activities of major CAZymes classes present in 10 grassland types on common glycoside linkages in the plant cell wall polysaccharides cellulose(a), xylan(b), xyloglucan(c), galactomannan(d), and rhamnogalacturonan(e). The red bold CAZymes classes have a relative abundance > 0.002%.


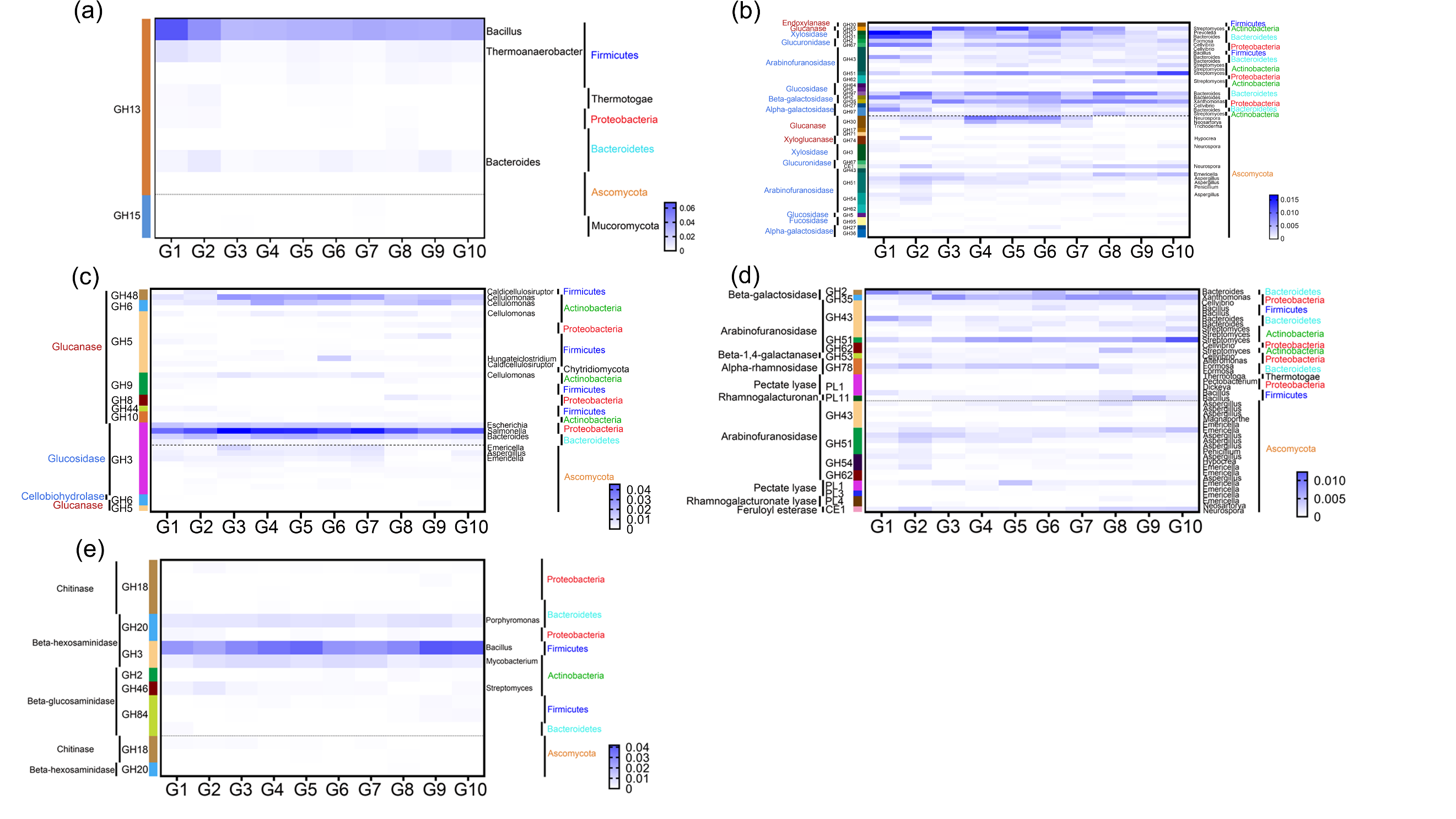


Figure S6. Major CAZymes classes and their corresponding microbial classification of polysaccharide degradation. a. amylase b. hemicellulose c. cellulase d. pectinase e. chitinase

Table S4 Topological properties of co-occurrence network of microbial communities.

| Network indexes |  |  |  |
| --- | --- | --- | --- |
| Nodes | 61346 | Positive edges (%) | 73.04% |
| Edges | 1447 | Negative edges (%) | 26.96% |
| R^2^of power-law | 0.918 | Positive edges of bacteria-bacteria (%) | 34.71% |
| ACC | 0.539 | Negative edges of bacteria-bacteria (%) | 15.70% |
| APL | 2.974 | Positive edges of bacteria-fungi (%) | 23.31% |
| diameter | 10 | Negative edges of bacteria-fungi (%) | 9.92% |
| Modularity | 0.474 | Positive edges of fungi-fungi (%) | 15.01% |
|  |  | Negative edges of fungi-fungi (%) | 1.34% |

Note: Abbreviations: ACC, average clustering coefficient; APL, average path length; Procrustes analysis, P < 0.001, M2= 0.256, 999 permutations; Mantel analysis, P < 0.05, r = 0.617

Table S5 Correlation analysis between physicochemical factors and biomass.

|  | MC | pH | BD | TC | TOC | TN | SON | NH_4_^+^-N | NO_3_^-^-N | TP | AP | TK | AK | UB | AB |
| --- | --- | --- | --- | --- | --- | --- | --- | --- | --- | --- | --- | --- | --- | --- | --- |
| MC |  |  |  |  |  |  |  |  |  |  |  |  |  |  |  |
| pH | -0.852** |  |  |  |  |  |  |  |  |  |  |  |  |  |  |
| BD | -0.872** | 0.675** |  |  |  |  |  |  |  |  |  |  |  |  |  |
| TC | 0.950** | -0.724** | -0.794** |  |  |  |  |  |  |  |  |  |  |  |  |
| TOC | 0.957** | -0.748** | -0.799** | 0.998** |  |  |  |  |  |  |  |  |  |  |  |
| TN | 0.888** | -0.802** | -0.876** | 0.826** | 0.832** |  |  |  |  |  |  |  |  |  |  |
| SON | 0.930** | -0.753** | -0.886** | 0.912** | 0.914** | 0.96** |  |  |  |  |  |  |  |  |  |
| NH_4_^+^-N | 0.837** | -0.779** | -0.765** | 0.819** | 0.829** | 0.833** | 0.842** |  |  |  |  |  |  |  |  |
| NO_3_^-^-N | -0.176 | -0.09 | 0.052 | -0.337 | -0.321 | -0.087 | -0.217 | -0.012 |  |  |  |  |  |  |  |
| TP | 0.903** | -0.873** | -0.792** | 0.872** | 0.881** | 0.861** | 0.861** | 0.892** | 0.009 |  |  |  |  |  |  |
| AP | 0.893** | -0.794** | -0.791** | 0.881** | 0.883** | 0.847** | 0.865** | 0.882** | -0.067 | 0.916** |  |  |  |  |  |
| TK | -0.659** | 0.505** | 0.663** | -0.63** | -0.635** | -0.655** | -0.715** | -0.487** | 0.148 | -0.562** | -0.651** |  |  |  |  |
| AK | -0.402* | 0.214 | 0.463** | -0.394* | -0.379* | -0.444* | -0.453* | -0.112 | 0.299 | -0.253 | -0.345 | 0.661** |  |  |  |
| UB | 0.905** | -0.745** | -0.801** | 0.857** | 0.862** | 0.905** | 0.94** | 0.766** | -0.322 | 0.793** | 0.818** | -0.682** | -0.446* |  |  |
| AB | 0.346 | -0.387* | -0.306 | 0.186 | 0.192 | 0.25 | 0.22 | 0.249 | 0.408* | 0.217 | 0.277 | -0.142 | -0.122 | 0.227 |  |

Table S6 The co-occurrences of the top 20 dominant genera with GH94.

| Node1 | Node2 | Correlation | P_value |
| --- | --- | --- | --- |
| g__Solirubrobacter\|p__Actinobacteria | GH94 | -0.757575758 | 0.0111434 |
| g__Rubrobacter\|p__Actinobacteria | GH94 | -0.963636364 | 7.32E-06 |
| g__Conexibacter\|p__Actinobacteria | GH94 | -0.878787879 | 0.0008139 |
| g__Sphingomonas\|p__Proteobacteria | GH94 | 0.818181818 | 0.0038149 |
| g__unclassified_o__Solirubrobacterales\|p__Actinobacteria | GH94 | -0.854545455 | 0.0016368 |
| g__Phycicoccus\|p__Actinobacteria | GH94 | 0.672727273 | 0.0330412 |


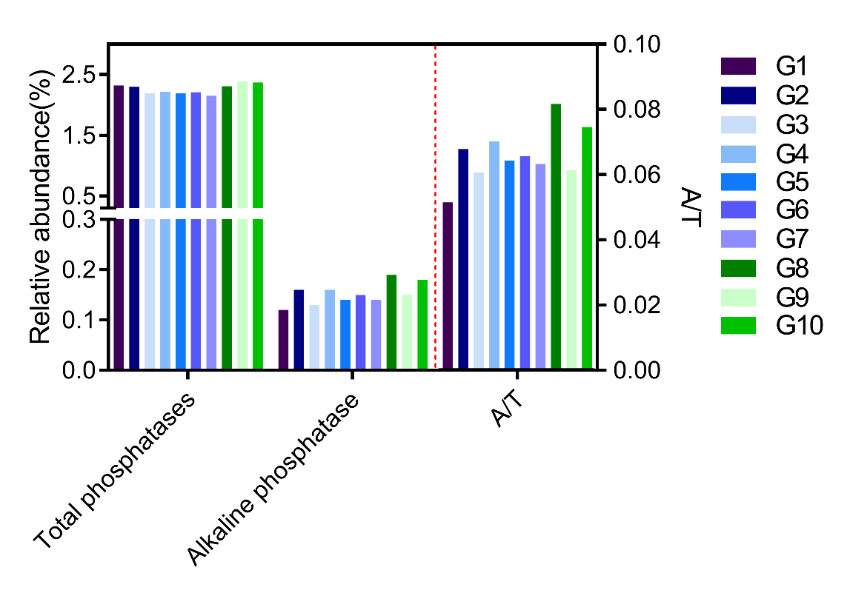


Figure S7. The relative abundance (>1%) of phosphatases.
